# Experimental Biodiversity Enrichment in Oil-Palm-Dominated Landscapes in Indonesia

**DOI:** 10.3389/fpls.2016.01538

**Published:** 2016-10-17

**Authors:** Miriam Teuscher, Anne Gérard, Ulrich Brose, Damayanti Buchori, Yann Clough, Martin Ehbrecht, Dirk Hölscher, Bambang Irawan, Leti Sundawati, Meike Wollni, Holger Kreft

**Affiliations:** ^1^Department of Systemic Conservation Biology, J.F. Blumenbach Institute for Zoology and Anthropology, Georg-August-University GöttingenGöttingen, Germany; ^2^Biodiversity, Macroecology and Biogeography, Georg-August-University GöttingenGöttingen, Germany; ^3^German Centre for Integrative Biodiversity Research Halle-Jena-LeipzigLeipzig, Germany; ^4^Institute of Ecology, Friedrich Schiller University JenaJena, Germany; ^5^Department of Plant Protection, Bogor Agricultural UniversityBogor, Indonesia; ^6^Centre for Environmental and Climate Research, Lund UniversityLund, Sweden; ^7^Department of Crop Sciences, Agroecology, Georg-August-University GöttingenGöttingen, Germany; ^8^Silviculture and Forest Ecology of the Temperate Zones, Georg-August-University GöttingenGöttingen, Germany; ^9^Tropical Silviculture and Forest Ecology, Georg-August-University GöttingenGöttingen, Germany; ^10^Faculty of Forestry, University of JambiJambi, Indonesia; ^11^Department of Forest Management, Faculty of Forestry, Bogor Agricultural UniversityBogor, Indonesia; ^12^Department of Agricultural Economics and Rural Development, Georg-August-University GöttingenGöttingen, Germany

**Keywords:** biodiversity-ecosystem functioning, tree planting, ecological restoration, ecosystem services, applied nucleation, agroforestry

## Abstract

Tropical biodiversity is threatened by the expansion of oil-palm plantations. Reduced-impact farming systems such as agroforests, have been proposed to increase biodiversity and ecosystem functioning. In regions where oil-palm plantations already dominate the landscape, this increase can only be achieved through systematic ecological restoration. However, our knowledge about the underlying ecological and socio-economic processes, constraints, and trade-offs of ecological restoration in oil-palm landscapes is very limited. To bridge this gap, we established a long-term biodiversity enrichment experiment. We established experimental tree islands in a conventional oil-palm plantation and systematically varied plot size, tree diversity, and tree species composition. Here, we describe the rationale and the design of the experiment, the ecosystem variables (soil, topography, canopy openness) and biotic characteristics (associated vegetation, invertebrates, birds) of the experimental site prior to the establishment of the experiment, and initial experimental effects on the fauna. Already one year after establishment of the experiment, tree plantings had an overall positive effect on the bird and invertebrate communities at the plantation scale. The diversity and abundance of invertebrates was positively affected by the size of the tree islands. Based on these results, we expect a further increase of biodiversity and associated ecological functions in the future. The long-term interdisciplinary monitoring of ecosystem variables, flora, fauna, and socio-economic aspects will allow us to evaluate the suitability of tree islands as a restoration measure. Thereof, guidelines for ecologically improved and socio-economically viable restoration and management concepts could be developed.

## Introduction

A major driver of the current biodiversity crisis in South-East Asia is the large-scale transformation of natural rainforest into simplified production systems such as oil palm (Fitzherbert et al., 2008; Immerzeel et al., 2014). As a consequence of the resulting dramatic losses of biodiversity, losses in ecosystem functioning are expected (Sodhi et al., 2004; Wilcove et al., 2013; Edwards et al., 2014) that can disproportionally exceed the loss in species diversity (Barnes et al., 2014). The degradation of important ecosystem functions such as pollination success or the impairment of soil fertility and water quality also puts human well-being at risk (Cardinale et al., 2012; Dislich et al., 2016).

Besides the importance of protecting tropical forests for biodiversity conservation, integrating biodiversity conservation into the management of existing large-scale oil-palm plantations seems imperative (Koh et al., 2009; Foster et al., 2011; Luskin and Potts, 2011; Teuscher et al., 2015). Designer plantation landscapes in which agroforestry zones buffer the natural vegetation from monoculture plantations have been proposed as one strategy to satisfy livelihood needs while increasing biodiversity and ecological functions (Koh et al., 2009). By enhancing the habitat complexity, the negative environmental impacts of intensively managed cash-crop production systems such as oil palm could be mitigated. Currently, institutions like the Roundtable for Sustainable Palm Oil (RSPO) focus on non-deforestation policy, conservation of large expanses of high valuable habitat, and threatened species (RSPO, 2013). However, in a region where most forest is lost (Margono et al., 2014) and where species diversity in the agricultural landscape is declining (Fitzherbert et al., 2008), options for conservation and reasonable landscape planning are already limited. Restoring habitat heterogeneity at local and landscape scales might thus be an option to maintain or even enhance biodiversity in oil-palm landscapes (Azhar et al., 2011).

Planting native trees has been considered a restoration measure to increase biodiversity (Chazdon, 2008). Planted tree islands can act as focal areas of recovery, or recruitment nuclei, and may initiate natural succession inside the islands and in its surroundings, as dispersers are attracted and establishment of new plant recruits is facilitated (sensu Yarranton and Morrison, 1974; Corbin and Holl, 2012). Such nuclei were found to have similar effects on biodiversity compared to tree plantings over large areas but are more cost-effective (Zahawi et al., 2013). Even small tree islands can act as recruitment nuclei as they increase bird activity and hence seed rain (Cole et al., 2010). For instance, seedling species richness was increased within a short period and seedling establishment was facilitated due to a more favorable microclimate in experimental tree islands in Honduras (Zahawi and Augspurger, 2006). Most restoration planting experiments took place in abandoned agricultural land, pastures, and logged-over forests (Zahawi and Augspurger, 2006; Cole et al., 2010; Hector et al., 2011), but tree islands were also suggested to enrich biota in agricultural landscapes (Rey Benayas et al., 2009). To date there is no consensus on which is ecologically and economically the most effective tree island size and how to transfer insights from island biogeography into a landscape context (Mendenhall et al., 2014).

**Box 1. Oil palm polycultures.**

In West Africa and Brazil, smallholders traditionally practice extensive oil-palm-based agroforestry to make up their livelihood. In South-East Asia, however, mainly high-productive, profit-maximizing monocultures dominate the landscapes (Corley and Tinker, 2003). Nevertheless, in all growing areas some smallholders intercrop oil palm seedlings with non-permanent food crops like maize, manioc, yam, cocoyam, soy bean, or cassava to bridge the income gap until the oil palms start fruiting (Lal et al., 1992; Okpala, 1995; Salako et al., 1995; Erhabor and Filson, 1999; Corley and Tinker, 2003). This, however, contributes little to a more heterogeneous structure which would benefit biodiversity (Phalan et al., 2009; Foster et al., 2011).

In a few experiments, oil palm was intercropped with trees, thereby creating permanent agroforests: In oil palm-rubber mixtures, negative effects due to light competition were reported for both species (Corley and Tinker, 2003). Oil palm–teak mixtures resulted in lower oil palm yields but enhanced teak performance (Chia, 2011). No yield depression from oil palms was noticed when intercropped with cacao [Lee and Kasbi, 1980 (Malaysia), Amoah et al., 1995 (Ghana)], and in Nigeria, cacao yields were even higher when planted under oil palms (Egbe and Adenikinju, 1990). In Indonesia, native tree species, including *Aquilaria malaquensis* and *Shorea* sp., proved to grow well under oil palms (Muryunika, 2015). In our study region, in Jambi province, Sumatra, Indonesia, management intensity of smallholdings varies, as around 50% of the farmers retain trees in their plantation, which benefits biodiversity but results in oil-palm revenue penalties (Teuscher et al., 2015); only few farmers intentionally plant trees, i.e., intercropping or along the borders (Muryunika, 2015). Despite many smallholders being interested in enriching their plantations with other trees, there is neither an approved system with specific implication guidelines nor is there any knowledge about the ecological and socio-economic costs and benefits of an oil-palm-based agroforestry.

To our knowledge, restoration efforts have rarely been made in an existing plantation; empirical support on how oil palm performs in polyculture comes from a few studies of intercropping systems (see **Box 1**). Furthermore, there is not much knowledge on how biodiversity enrichment affects biodiversity and socio-economics.

**FIGURE 1:**
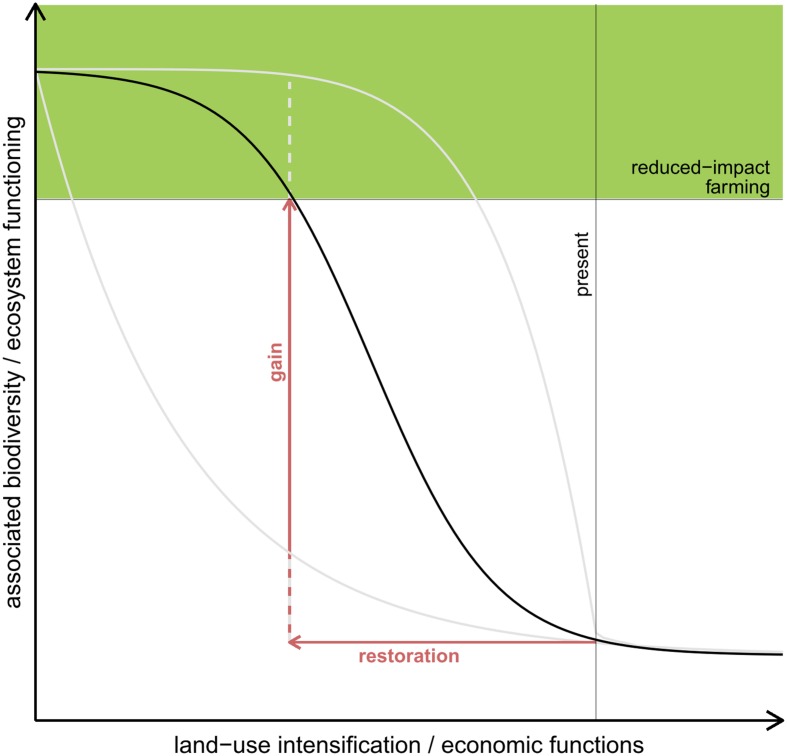
**Possible scenarios of changes in biodiversity and ecosystem functions (BEF) as a consequence of land-use intensification assuming a negative and non-linear relationship between land-use intensification and BEF**. Consequently, there is space for restoration measures in order to enhance ecosystem functioning while still allowing for profitable land use. The loss in ecosystem functioning is supposed to be relatively slow with extensive land use (shaded area) but reaches a critical point once the buffer ability of the ecosystem is exhausted. Further land-use intensification will then result in a severe decline in ecosystem functioning. The optimal trade-off situation between nature conservation and land use would be when intensification is stopped before the critical point is reached. In oil-palm-dominated landscapes, however, this point might already be exceeded, as BEF are severely degraded in oil-palm systems (Barnes et al., 2014; Kotowska et al., 2015; Dislich et al., 2016). To move back to the critical point, diverse habitats have to be restored.

Numerous experiments investigating the relationship between biodiversity and ecosystem functioning (BEF) have shown that adding a few species can already lead to a disproportionate increase in ecosystem functioning (Balvanera et al., 2006; Cardinale et al., 2006, 2012; Quijas et al., 2010). This suggests that adding species to an extremely depauperate system can result in relatively high gains in ecosystem functioning (**Figure 1**), both as the added species directly contribute to enhanced ecosystem functioning and increase the heterogeneity in resources and structure that could attract other organisms (Tews et al., 2004).

Recently, insights from BEF research found their way into restoration ecology (Aerts and Honnay, 2011). However, most of the findings related to BEF have been obtained from small-scale studies in temperate grasslands and a number of large-scale tree planting experiments have only lately been established (Scherer-Lorenzen et al., 2005; Verheyen et al., 2015); six BEF experiments with trees are located in the tropics (Petit and Montagnini, 2006; Moreira et al., 2014; Verheyen et al., 2015). Early results from these experiments suggest that diverse plantings lead to a higher increase in ecosystem functions compared to monocultures (e.g., Potvin and Gotelli, 2008).

The knowledge gaps regarding the ecological consequences of restoration via enrichment plantings in oil-palm landscapes go along with limited knowledge about the impacts on the local socio-economy. In some parts of South-East Asia, the area of oil palms managed by smallholders is currently more rapidly increasing than the area managed by large estates (Euler et al., 2015; Gatto et al., 2015), resulting in a growing number of households depending on palm-oil production. Therefore, it is essential to develop strategies that, at least partly, compensate potential income losses due to restoration plantings. In this regard, crop diversification may be one option, as it acts as insurance, e.g., as a buffer for world-market price-fluctuation, climate change impacts, or possible pest attacks (Lin, 2011). Additionally, it can have benefits in the short-term, e.g., by the provision of raw material or food for self-consumption or also financially through more efficient use of the available arable land. Further, enhanced biodiversity can improve the provision of ecosystem services that are beneficial to oil-palm management. Biological pest control, pollination, and litter decomposition (and thus soil fertility) are among the most important ecosystem services for productive oil-palm management (Foster et al., 2011) and can directly benefit the farmers’ income (Tscharntke et al., 2011). This might raise the willingness to accept and adopt novel management forms.

We hypothesize that restoration plantings have the potential to help enhance biodiversity and ecosystem functions in impoverished landscapes whilst minimizing financial losses (**Figure 1**). Clear management strategies for restoration of intensively managed oil-palm landscapes toward ecologically improved and at the same time economically viable systems, however, are yet to be developed. Several questions have to be considered in this context: how many species need to be planted to gain a significant increase in ecosystem functioning? Which species composition and island size is the most effective? What are the trade-offs between BEF and socio-economics?

Here, we (1) present the design of a biodiversity enrichment experiment (BEE) in a monoculture oil-palm landscape (2) measure heterogeneity in the oil-palm plantation as a baseline for the experiment (3) describe abiotic and biotic characteristics of the plantation and (4) present first results of the effects of the enrichment plantings on birds and invertebrates 1 year after the establishment of the experiment.

## Materials and Methods

### Study Site

**FIGURE 2:**
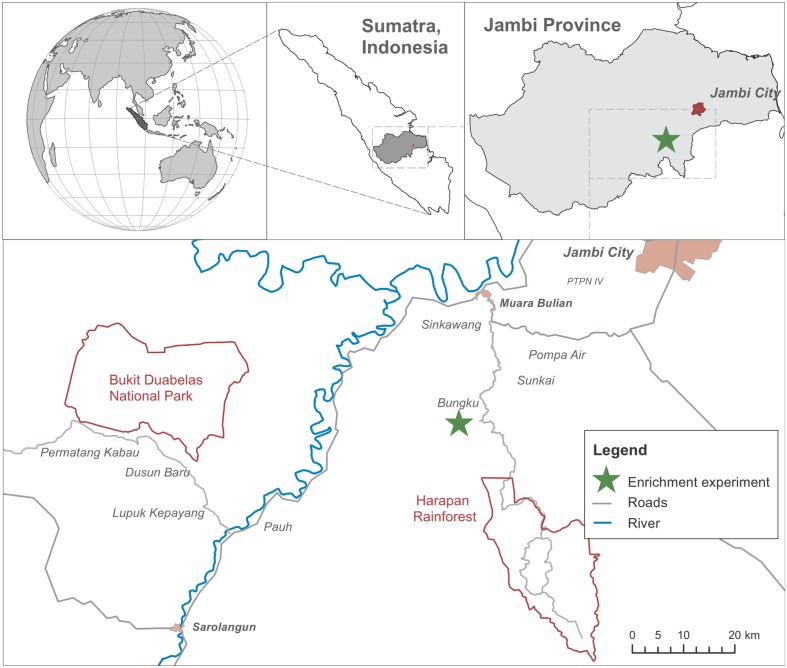
**Map of the study area (Drescher et al., 2016; modified)**. The green star indicates the location of the study site where the biodiversity enrichment experiment (EFForTS-BEE) was established.

Our enrichment planting experiment was established on an oil-palm plantation of PT. Humusindo Makmur Sejati (01.95° S and 103.25° E, 47 ± 11 m a.s.l.) near Bungku village in the lowlands of Jambi province, Sumatra (**Figure 2**). The climate is humid tropical, with a mean temperature of 26.7 ± 1.0°C and an annual rainfall of 2235 ± 385 mm (1991–2011; measured at Jambi Sultan Thaha airport of the Meteorological, Climatological and Geophysical Agency). The dominant soil type in the region is loamy Acrisol (Allen et al., 2015). Dipterocarp-dominated lowland rainforests are the primary natural vegetation (Whitten et al., 2000; Laumonier et al., 2010).

The planting of oil palms in the plantation started in 2001 and, according to satellite images, ended approximately in 2006 or 2007 (Google Earth, 2015), leading to an inhomogeneous age structure of ca. 6–12 years. Oil palms are planted in 9 m × 9 m triangular grid resulting in ca. 143 oil palms per ha. In 2014, the average oil palm yield on the plantation was 22.74 metric tons of fresh fruit bunches ha^-1^ y^-1^. The management of the plantation comprises fertilizer application [230 kg N (Urea), 196 kg P (Triple Superphosphate and rock phosphate), 142 kg K (KCl), 54 kg Mg (Kieserite and Dolomite), and 0.79 kg B (Borax), all in ha^-1^ year^-1^; additionally S ((NH_4_)_2_SO_4_), Si (Zeolite), and Ca], regular manual weeding of the understory, and removal of epiphytes. Herbicides are only rarely used when there are not enough workers available for manual weeding. Livestock farming is also practiced on the plantation.

### The Biodiversity Enrichment Experiment (EFForTS-BEE)

**FIGURE 3:**
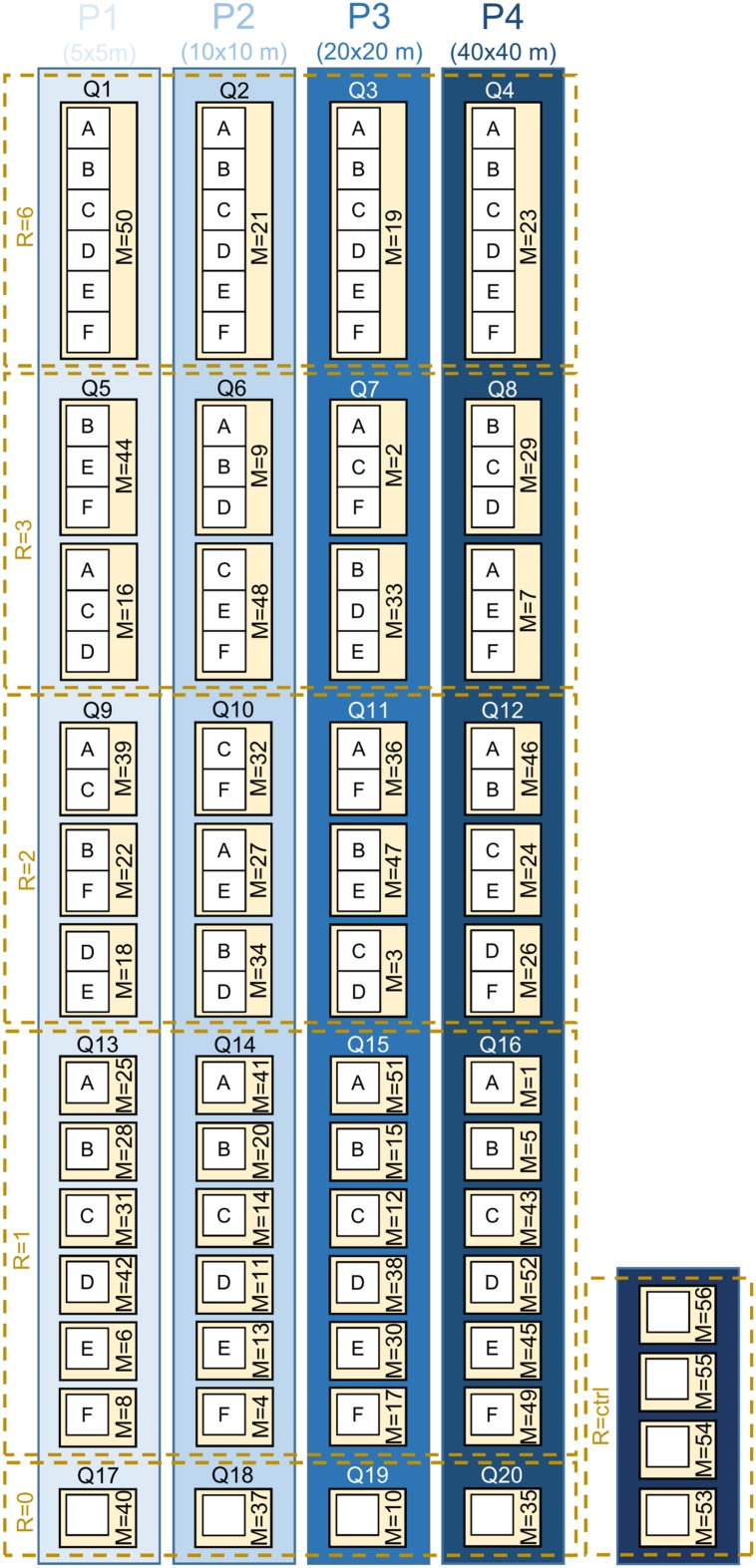
**Schematic overview of the experimental plots adopting a random partitions design (see Bell et al., 2009 for detailed information)**. ‘P’ stands for the four partitions that differ in plot size (P1 = 5 × 5 m, P2 = 10 × 10 m, P3 = 20 × 20 m, P4 = 40 × 40 m). Each partition ‘P’ is divided into five blocks ‘Q’ (Q1–Q20), one per tree diversity level ‘R’ (*R* = 0/1/2/3/6). Within each of these blocks, each species is randomly drawn from the species pool without replacement. Between the plots ‘M’ (*M* = 1–52; numbers represent the individual Plot IDs), no repetition of the species composition was allowed (tree species: A, *Parkia speciosa*, Fabaceae; B, *Archidendron pauciflorum*, Fabaceae; C, *Durio zibethinus*, Malvaceae; D, *Dyera polyphylla*, Apocynaceae; E, *Peronema canescens*, Lamiaceae; F, *Shorea leprosula*, Dipterocarpaceae). Additionally, there are four control plots (*R* = ctrl, *M* = 53–56) of the same size (10 m × 10 m). Trees were planted on plots with *R* = 1/2/3/6, but not on plots with *R* = 0/ctrl. A special experimental management (stop of herbicide/pesticide/fertilizer application and stop of weeding 2 years after establishment) is applied on the plots *M* = 1–52; plots *M* = 53–56 are managed-as-usual. The actual spatial arrangement of the plots in the plantation was random; plots were not aggregated according to partitions, blocks, or diversity level.

**FIGURE 4:**
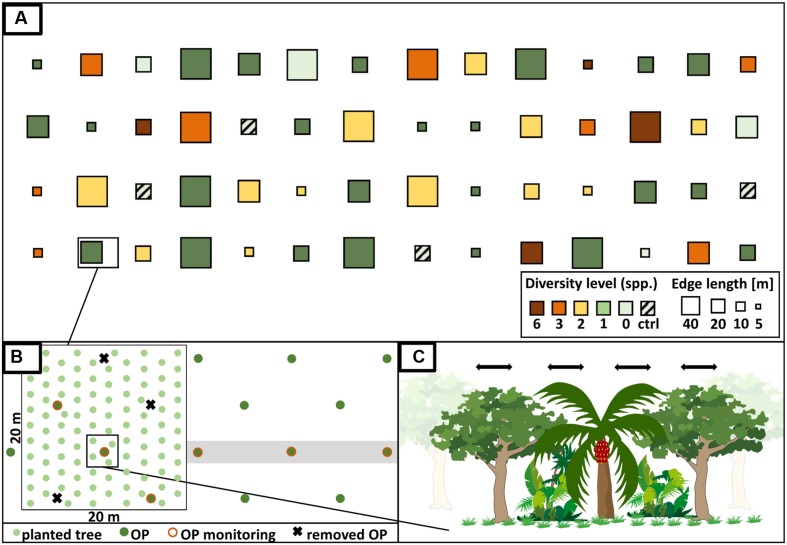
**(A)** Design of the biodiversity enrichment experiment (EFForTS-BEE). Tree islands with systematically varying tree diversity (diversity level of 0, 1, 2, 3, and 6), identity and composition as well as plot size (5 m × 5 m, 10 m × 10 m, 20 m × 20 m, 40 m × 40 m) and species composition were established adopting a random partitions design (Bell et al., 2009). Partitions differ in their plot size and are subdivided into blocks of varying tree diversity levels. At each level of diversity, each tree species is represented exactly once. On plots with treatment (diversity level 0–6), a special management is applied (stop of fertilizer and pesticide application; manual weeding). Additionally, the experiment includes four control plots without treatment and with management-as-usual. In total, the experiment comprises 56 plots. **(B)** Oil palms (OP) were cut on the plot with treatments in order to enhance light conditions. Trees were planted in a 2 × 2 m grid. Perpendicular to each plot, three oil palms were selected to monitor services and disservices of the tree islands on surrounding oil palms. **(C)** Planted trees interact/compete with each other as well as with the oil palms (Ian Image, 2015; modified). Manual weeding will stop after two years to allow for natural succession.

We established a large-scale, long-term BEE within a monoculture oil-palm landscape as a sub-project of the EFForTS^1^ [Ecological and socio-economic functions of tropical lowland rainforest transformation systems (Sumatra, Indonesia)] research initiative that investigates the impacts of transforming lowland rainforest into land-use systems such as oil-palm plantations (Drescher et al., 2016). Tree islands of varying species diversities and compositions were established with a minimum distance of 85 m between them. Across experimental plots, we varied the diversity and identity of the tree species planted, adopting a random partitions design (see Bell et al., 2009 for detailed information) (**Figure 3**). The design allows disentangling the linear effects of plot size, tree diversity, and non-linear effects of tree species composition. This approach analyzes gradients using stepwise linear regression models rather than comparing distinct groups. Thus, a full-factorial setup, which is usually not feasible, is not needed. The experiment comprises four partitions that differ in their plot size (5 m × 5 m, 10 m × 10 m, 20 m × 20 m, 40 m × 40 m). Each partition is divided into five blocks, one per tree diversity level (0, 1, 2, 3, and 6 species). Within each of these blocks, each species is randomly drawn from the species pool without replacement. Each species is thus selected exactly once at each diversity level and species compositions are random, with the restriction that no repetition across all plots was allowed (**Figure 3**). Additionally, there are four control plots of the same size without any experimental treatment and management-as-usual. This results in a total of 56 plots (Appendix Table 1). The spatial arrangement of the plots in the plantation was random; i.e., plots were not aggregated according to partitions, blocks, or diversity level (**Figure 4A**).

We selected six native multi-purpose tree species including three trees grown mainly for fruits (*Parkia speciosa*, Fabaceae; *Archidendron pauciflorum*, Fabaceae; *Durio zibethinus*, Malvaceae), two species used for timber (*Peronema canescens*, Lamiaceae; *Shorea leprosula*, Dipterocarpaceae), and one species which produces natural latex (*Dyera polyphylla*, Apocynaceae). To enhance the light availability in the experimental plots by ca. 40%, we removed selected oil palms prior to tree planting (not on the control plots in all sizes and not on the 5 m × 5 m plots which are in between oil palms).

In December 2013, trees were planted in a 2 m grid in alternating rows in north-south direction. On mixed-species plots, trees of the same species were planted as far away as possible from one another. We planted six trees on the 5 m × 5 m plots, 25 trees on the 10 m × 10 m plots, 100 trees on the 20 × 20 m plots and 400 on the 40 × 40 m plots. The total number of planted trees was 6354.

To enhance the establishment success of the trees, we applied inorganic (19 kg N, 8 kg P, 6 kg K, 3 kg Mg, all in ha^-1^) and organic (11 kg N, 7 kg P, 10 kg K, 4 kg Mg, 20 kg Ca, all in ha^-1^) fertilizer once inside the planting holes before we planted the trees on plots with diversity level 1–6 (note that this fertilizer treatment was not applied on 0-diversity plots but only on plots with trees planted).

The management of all experimental plots (diversity level 0–6) comprises manual weeding to prevent weeds from overgrowing the planted saplings (approximately every 3 months) but will, except for small circles around the trees on plots with diversity level 1–6, be stopped after two years to allow succession (**Figure 4C**). The application of fertilizer, herbicide and pesticides inside plots stopped after planting. Fences around plots with diversity level 0–6 protect the plots, and particularly the planted trees, from damage by mammals. Dead trees were replaced during the first year after establishment.

The long-term monitoring of the EFForTS-BEE includes recording (a) the ecosystem variables (soil, canopy cover, surrounding matrix), (b) plants (tree mortality and growth, understory vegetation, seed rain, herbivory), (c) animals (bird and invertebrate community), and (d) socio-economics (oil-palm yields, benefits from the planted trees, incentive for enrichment planting).

In order to quantify potential ecological services or disservices from enrichment plantings on the surrounding oil palms, individual yield of three oil palm individuals in perpendicular direction from the plot are monitored (**Figure 4B**). Additionally, the yield of each oil palm inside the plot is measured as part of the long-term monitoring.

### Sampling of Environmental Variables, Flora, and Fauna

A baseline survey of the environment, vegetation, birds, and invertebrates was conducted in October 2013 prior to the establishment of EFForTS-BEE. In October 2014, bird and invertebrate surveys were repeated. Due to heavy disturbance in the ground vegetation layer during tree planting in December 2013, we did not repeat the vegetation survey; the data from 2013 would not have been comparable to the situation in 2014.

In each plot, slope was measured along all four plot edges and diagonal from the southwestern to the northeastern corner using a Vertex measuring instrument (Haglöf). We used the maximum slopes [in °] for further analyses.

Soil composite samples were taken on each plot at 0–10 cm depth. Samples were then oven-dried (40°C, 48 h), ground and sieved (2 mm) for further analyses. Soil texture (20 g of soil) was analyzed using pipette methods. Soil organic C was measured with a CN analyzer (MT-1000, Yanako, Kyoto, Japan). Ten grams of dry soil were diluted in 25 ml H_2_O to determine the pH-value. For bulk density (dry weight [g]/cylinder volume [cm^3^]) analysis, a standardized soil volume (250 cm^3^) was taken in 5–10 cm depth, oven dried (105°C, 48 h), and immediately weighed.

On each plot, we established one randomly placed 2 m × 2 m subplot (random coordinates, X on south-north and Y on west-east axis with a minimum of 1.5 m distance to the plot edges). We estimated the percentage of bare soil, i.e., the area without any vegetation cover in the subplot.

Prior to oil-palm cutting, hemispherical photographs were taken at the subplot-center of each plot using a Canon 700D camera and a fisheye lens (SIGMA 4.5/2.8 EX DC HSM) and different exposure settings (see Beckschäfer et al., 2013). The gap fraction was calculated using the best picture per plot (maximum exposure time without being over-exposed) using ‘ImageJ’ (version 1.48v). One year after the establishment, hemispherical photographs were repeated, but covered the whole plot area with varying number of spots depending on the plot size (one spot in 5 × 5, one in 10 × 10, three in 20 × 20, seven in 40 m × 40 m) and gap fraction was calculated as means per plot to control for inhomogeneous canopy densities due to oil-palm cutting.

Individual-based vegetation surveys of all vascular plants ≥5 cm were conducted on each subplot. Herbarium specimens (Collection Numbers AG01-AG167, deposition and identification in SEAMEO BIOTROP institute, Bogor, Indonesia) were collected for plant identification.

Point counts of birds took place from 6 am to 10.30 am when weather conditions were appropriate (no rain). Birds within a 75 m radius around each plot center were recorded visually and acoustically using 15-min point counts (following the taxonomy of MacKinnon et al., 1993). Each sampling point was visited twice. For each species, we recorded the maximum number of individuals present simultaneously on the plot. For all recorded species, body mass was obtained from the literature (Wilman et al., 2014) to calculate bird biomass. Species were assigned to five trophic groups (insectivores, frugivores/nectarivores, herbivores/granivores, piscivores/scavengers, omnivores) and to their main natural habitat (primary and old secondary forest interior; forest gaps, edges or upper canopy; little wooded and cultivated areas). Information on diet was obtained from Wilman et al. (2014). Information on habitat was also taken from the literature (Thiollay, 1995; Pappas, 2001; Beukema et al., 2007; Robson, 2015; Yosef et al., 2015).

We extracted invertebrates from the leaf-litter (LL) by sieving the LL from 1 m^2^ within each subplot through a coarse sieve (mesh width = 2 cm) (see Digel et al., 2014; Ott et al., 2014). Invertebrates in the herb layer (HL) were sucked in from 1 m^2^ within each subplot using a modified vacuum cleaner. Specimens were stored in 70% ethanol, identified to family level, and assigned to trophic groups (predators, omnivores, herbivores, and detritivores). Individual body length (accuracy of 0.1 mm) was converted to fresh body mass using length-mass allometric functions (Appendix Table 2) and, where necessary, dry mass-fresh mass relationships from the literature (Appendix Table 3). We summed up the fresh masses of the individuals to calculate the total biomass per plot. Samples were collected based on collection permit no. 648/KKH-2/2014 and 15/KKH-2/2013, recommended by LIPI and issued by the Ministry of Forestry (PHKA).

### Statistical Analysis

We conducted a principal component analysis (PCA) with the soil variables (texture, pH, C content, and bulk density; Appendix Table 4) to reduce their predominance in the set of site-condition variables (**Table 1**) to generalized trends, and used the scores of the first three PCA axes for further analyses.

**Table 1:** Ecosystem variables of the experiment.

Variable	Unit	Mean ± SD
Altitude	[m]	46.9 ± 10.5
Slope	[°]	8.6 ± 5.9
Bare soil	[%]	11.0 ± 10.6
Gap fraction_baseline_	[%]	14 ± 10.0
Gap fraction_year1_	[%]	27.5 ± 14.9
Oil palm trunk height	[m]	3.83 ± 0.6
Soil Bulk Density	[g/cm^3^]	1.09 ± 0.1
Sand	[%]	29.9 ± 12.6
Silt	[%]	40.5 ± 8.3
Clay	[%]	29.5 ± 8.3
pH (1:2.5 H_2_O)		3.97 – 4.11 – 5.3
C	[%]	2.18 ± 0.6

*Per variable, means of all plots are given with the standard deviation, except for the pH-value, where the full variable range is shown in addition to the mean. We show the gap fraction prior to cutting (baseline) and after cutting (year 1; mainly above the planted trees). Average oil palm height was derived from all plots (*N* = 31) where oil palms remained*.

To check for unintended systematic correlations between the site-condition variables and the experimental factors, we ran linear models with the site-condition and biotic variables (Appendix Table 5) as responses and ‘tree diversity’ and ‘plot size’ as predictors. Further, we investigated the spatial autocorrelation of the site-condition parameters using Moran’s I correlograms (standard deviate with 100 permutations, distance classes of 150 m) to test whether the site-condition variables in our plots are spatially dependent.

We calculated α-diversity as 1 – Simpson-index; β-diversity was calculated as 1 – Sørensen-index based on true abundance data (Legendre and De Cáceres, 2013) for all organism groups [vegetation (subplot), birds (75 m radius around plot center), LL invertebrates (subplot), HL invertebrates (subplot)]. We estimated species/family richness for each organism group using ‘Jackknife 2’ due to high mean evenness-values (vegetation: 0.67, birds: 0.84, LL invertebrates: 0.72, HL invertebrates: 0.82) (Brose et al., 2003).

We tested for the overall effect of tree planting by comparing the baseline survey and year one of the richness, abundance, and biomass of birds as well as LL and HL invertebrates generalized least square models and Tukey *post hoc* tests. We compared data from plots with diversity level 1–6 with data from plots with diversity level 0 and control plots.

Furthermore, we tested for the effect of tree diversity (levels of 1, 2, 3, and 6) and plot size (25, 100, 400, and 1600 m^2^; ln-transformed) on the difference in richness, abundance, and biomass of birds and LL/HL invertebrates in year one compared to the baseline survey, following the stepwise linear regression approach by Bell et al. (2009). We tested for linear, non-linear, and identity effects of plot size and tree diversity.

We investigated the effect of ‘plot size’ and ‘tree diversity’ on possible shifts in the relative proportions of invertebrate biomass and abundance within trophic compartments in year one compared to the baseline survey. The analyses were based on the community-weighted mean of the biomass and abundance of HL and LL invertebrates per plot. For the calculation, scores were assigned for trophic levels (herbivores, detritivores ‘0’; omnivores ‘0.5’; predators ‘1’), multiplied with the biomasses of the individuals, summed up per plot, and divided by the total biomass per plot. Community-weighted mean was modeled using a linear mixed model; ‘tree diversity,’ ‘plot size’, and its second order polynomial term (to test for non-linear effects of plot size) as well as ‘year’ entered the full model as predictors in a three-fold interaction. ‘Plot ID’ was included as a random effect. A backward selection of the full model was done to identify the most important predictors. All analyses were conducted in R (R Core Team, 2015) using the following packages: vegan (Oksanen et al., 2015), FD (Laliberté et al., 2014), ncf (Bjornstad, 2013), nlme (Pinheiro et al., 2015).

## Results

### Ecosystem Variables of the Plantation

Some site-condition baseline characteristics (topography, gap fraction, proportion of bare soil, soil texture, and soil carbon content) varied greatly among plots, while bulk density and soil pH were rather homogenous (**Table 1**; Appendix Table 4).

The first three PCA axes explained 80% of the variation in the measured soil characteristics (Appendix Figure 1). Soil texture (silt, sand and clay) contributed most to the first PCA axis; soil texture (clay), carbon content, and bulk density to the second; and soil pH to the third (Appendix Table 4).

The proportion of bare soil as well as silt and sand content are spatially dependent on short distances, clay and sand content on large distances, and soil pH in one medium-distance class with a low correlation coefficient (0.15) (Appendix Figure 2). We detected systematic relationships between the two experimental factors ‘tree diversity’ and ‘plot size’ with some site-condition and some biotic variables. However, the strengths of the effects were in all cases negligible (*R*^2^ values < 0.09) (Appendix Table 6).

### Flora and Fauna

We recorded a total of 92 plant species, 21 bird species, 87 LL, and 94 HL invertebrate families on the experimental plots (**Table 2**). Jackknife 2-estimated richness was substantially higher for plant species (157 species; 58.6% sample representativeness) and invertebrate families (LL/HL: 137/148 families; 63.5% sample representative in both groups) but not for birds (26 species estimated; 80.8% sample representativeness). These findings were consistent with species accumulation curves (Appendix Figure 3). The α-diversity was similar for all organism groups (0.62–0.76) (**Table 2**). The abundance based β-diversity ranged from 0.12 to 0.2 (**Table 2**).

**Table 2:** Species/family numbers of the four organisms groups monitored at the experimental sites in the baseline survey.

	Vascular plants	Birds	LL invertebrates	HL invertebrates
Total species/family richness	92 (species)	21 (species)	87 (families)	94 (families)
Estimated species/family richness	157	26	137	148
Mean species/family number per plot (± SD)	16.67 ± 4.55	4.42 ± 2.11	9.4 ± 5.76	11.6 ± 6.34
β-diversity	0.12	0.18	0.19	0.2
α-diversity, mean per plot (± SD)	0.76 ± 0.12	0.63 ± 0.19	0.62 ± 0.23	0.76 ± 0.13

*LL, leaf litter; HL, herb layer*.

#### Vegetation

Of the 92 plant morphospecies, 64 could be identified of which 25 were alien species (Appendix Table 7). The three most frequent species – *Clidemia hirta* (Melastomataceae) followed by *Asystasia gangetica* (Acanthaceae) and *Paspalum* cf. *conjugatum* (Poaceae) – were non-native species.

#### Birds

A total of 21 species were detected (Appendix Table 8). All species are listed as “least concern” (IUCN, 2015). Of the recorded individuals, 48.8% were insectivores, 35.5% frugivores/nectarivores, 7.2% omnivores, 2.8% herbivores/granivores, and 5.8% were piscivores/scavengers. The main natural habitat for 1.6% of the sampled individuals is primary and old secondary forest interior, for 7.5% forest gaps, edges or upper canopy, and for 90.9% little woods and cultivated areas.

#### Invertebrates

From the LL, 87 families (Appendix Table 9) were collected. The sampled individuals consisted of 24.8% predators, 61.2% omnivores, 1.8% herbivores, 9.7% detritivores, and 2.5% others. In the HL, 94 families were collected (Appendix Table 10). The invertebrates sampled consisted of 18.7% predators, 46% omnivores, 18.3% herbivores, 11.6% detritivores, and 5.4% others.

### Overall Effect of Tree Planting on the Bird and Invertebrate Community One Year after Establishment

#### Birds

We recorded 20 species (Appendix Tables 8 and 11), whereof 15 species where the same as in 2013 and five species were new. All species are listed as “least concern” (IUCN, 2015). Of the recorded individuals; 44.5% were insectivores, 31.7% frugivores/nectarivores, 2.3% omnivores, 16.5% herbivores/granivores, and 5.0% were piscivores/scavengers.

**FIGURE 5:**
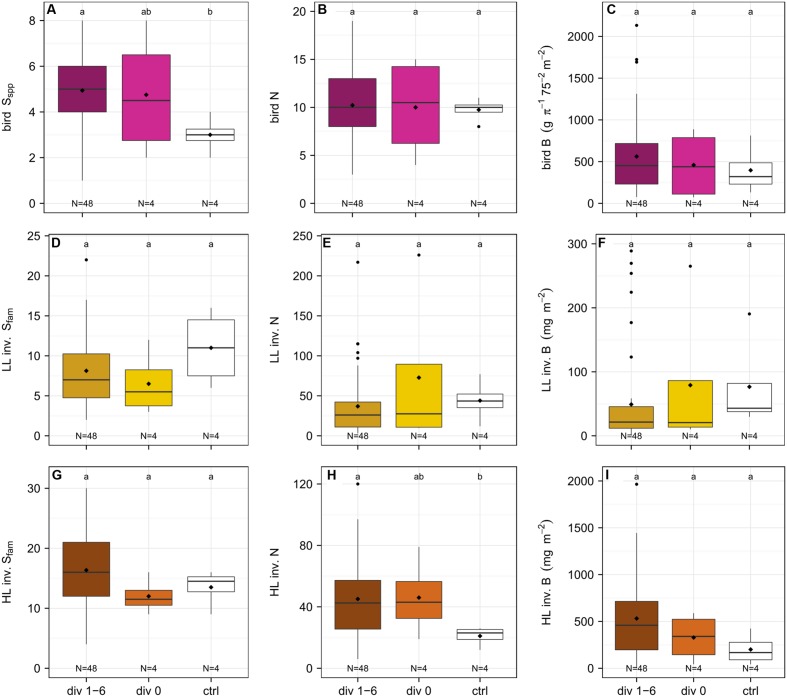
**Comparison of the richness (S_spp_, species level, S_fam_, family level), abundance (N) and biomass (B) of birds **(A–C)**, leaf-litter (LL) **(D–F)** and herb-layer (HL) **(G–I)** invertebrates (inv.) between plots with diversity level 1, 2, 3, and 6 (*N* = 48), plots with diversity level 0 (*N* = 4), and control plots (*N* = 4) one year after establishment**. Bird species richness and the abundance of HL invertebrates were significantly increased on plots with trees compared to control plots.

In year one of the experiment, bird species richness was significantly higher on plots with diversity level 1–6 as compared to the control plots (management-as-usual) (*p* < 0.001) but not different from plots with diversity level 0 (*p* > 0.05) (**Figure 5A**). Furthermore, there was no difference in richness between experimental plots and control plots (*p* > 0.05). The abundance and biomass of birds was not significantly affected by any experimental treatment (*p* > 0.05) (**Figures 5B,C**).

#### Invertebrates

A total of 74 families were collected in the LL (Appendix Tables 9 and 11) of which 48 were the same as in 2013, 26 were new, and 39 were not represented anymore. The sample comprised 17.1% predators, 70.7% omnivores, 3% herbivores, 7.3% detritivores, and 1.9% others. Family richness, abundance and biomass of the LL invertebrates did not differ between plots with diversity level 1–6, plots with diversity level 0, and control plots (*p* > 0.05) (**Figures 5D–F**).

In total, 105 families were collected in the HL (Appendix Tables 10 and 11). Compared to the year before, 58 families were the same, 47 were new, and 36 were not present anymore. The invertebrates consisted of 17.2% predators, 48% omnivores, 15.3% herbivores, 11.5% detritivores, and 8% others.

Herb layer invertebrates were significantly more abundant on experimental compared to the control plots (*p* < 0.01), but there was no significant difference in HL invertebrate abundance between plots with diversity level 0 and those with diversity level 1–6 (*p* > 0.05) (**Figure 5H**). Family richness and biomass were not affected by the experimental treatment (*p* > 0.05) (**Figures 5G,I**).

### Initial Effects of Tree Diversity and Plot Size on the Bird and Invertebrate Community

**FIGURE 6:**
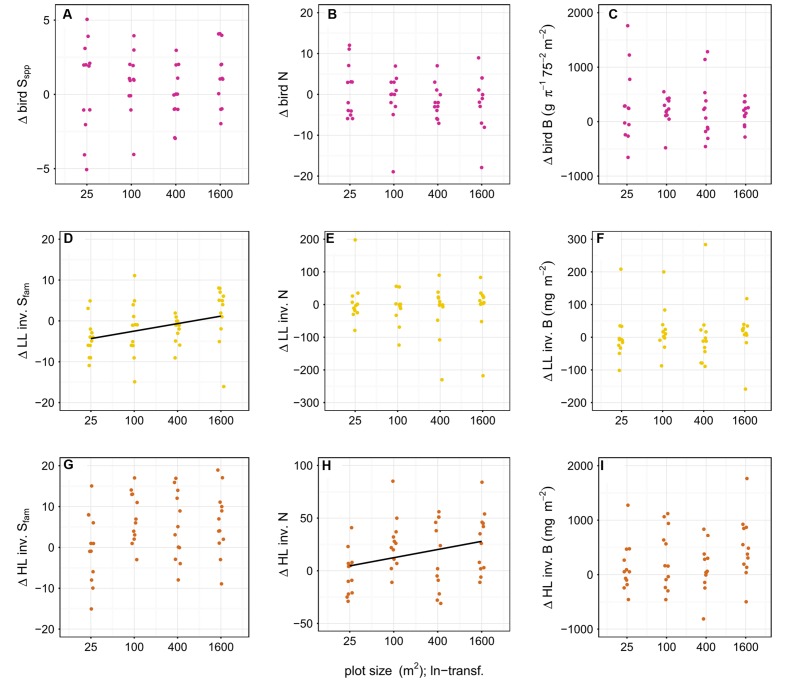
**Effect of plot size on the difference in richness (S_spp_ = species level, S_fam_ = family level), abundance (N) and biomass (B) of birds **(A–C)**, leaf-litter (LL) **(D–F)** and herb-layer (HL) **(G–I)** invertebrates (inv.) between year one and the baseline**. LL invertebrate family richness and HL invertebrate abundance was significantly positively related to plot size (indicated by a black line). Plot sizes (25, 100, 400, and 1600 m^2^) were ln-transformed for improved representation in the figure. To avoid overplotting of data points, we used the ‘jitter’ function in R (R Core Team, 2015).

**FIGURE 7:**
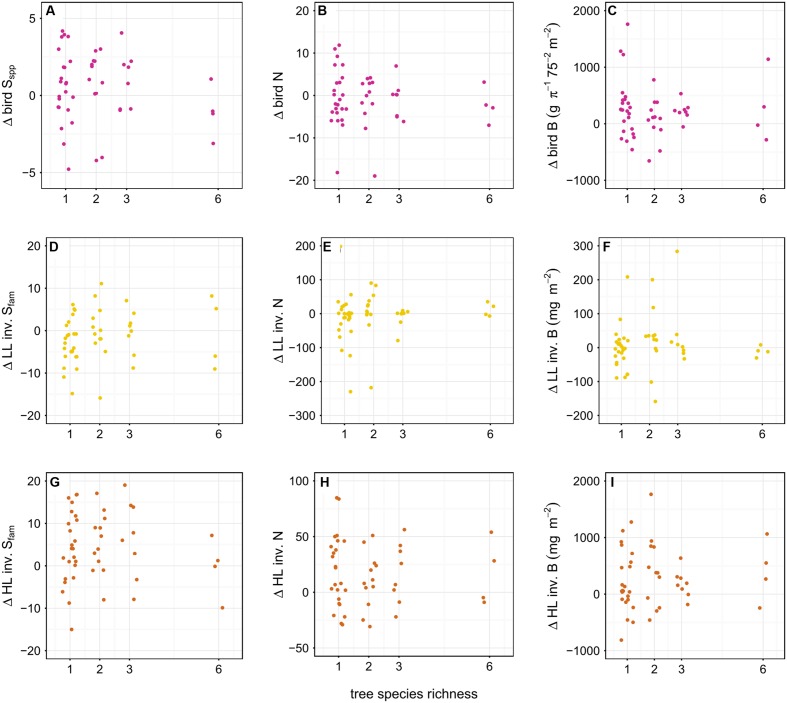
**Effect of tree diversity on the difference in richness (S_spp_ = species level, S_fam_ = family level), abundance (N) and biomass B of birds **(A–C)**, leaf-litter (LL) **(D–F)** and herb-layer (HL) **(G–I)** invertebrates (inv.) between year one and the baseline**. Birds were considered on species level, invertebrates on family level. There was no effect of tree diversity on any of the responses. To avoid overplotting of data points, we used the ‘jitter’ function in R (R Core Team, 2015).

We found a significantly positive effect of plot size on the difference in diversity of LL family richness (*p* < 0.05) and the difference in abundance of HL invertebrates in year one compared to the baseline (*p* < 0.05) (**Figure 6**); Tree diversity, however, did not affect the difference in richness, abundance, and biomass of birds and invertebrates (*p* > 0.05) (**Figure 7**).

### Shifts of Invertebrate Biomass and Abundance Within Trophic Compartments

We found non-significant effects (*p* > 0.05) of tree planting (factor ‘year’), plot size (plot size: year), and tree diversity (tree diversity: year) on the difference of the community-weighted mean trophic index and abundance of LL and HL invertebrates between year one and the baseline. This suggests that changes in the proportion of invertebrate biomass and abundance within the trophic compartments are likely to be driven by other than the experimental factors.

## Discussion

By experimentally investigating plot size and tree diversity – two key factors in a restoration context – EFForTS-BEE aims at shedding light on the ecological and socio-economic processes associated with ecological restoration of oil-palm landscapes. The controlled experimental design of EFForTS-BEE allows us to investigate the underlying mechanisms of enrichment plantings.

Our study site in Jambi province, Sumatra, is ideal for studying the long-term effects of enrichment plantings. We have chosen a medium-scale oil-palm plantation for the experiment that is embedded in an intensively oil-palm and rubber-dominated landscape. The average oil palm yield of 22.74 metric tons of fresh fruit bunches ha^-1^ y^-1^ is on the higher end compared to smallholder plantations in the region (18.02–23.72 t ha^-1^ y^-1^, Kotowska et al., 2015). This might be explained by a higher and more diverse fertilizer use compared to smallholders (Hassler et al., 2015; Kotowska et al., 2015). The management might hence be similar to other mid- or large-scale oil-palm plantations. Furthermore, the diversity of plants, birds, and invertebrates at the study site is comparable to and thus representative of the diversity in other oil-palm plantations in the region (Appendix Table 12) (Drescher et al., 2016).

The results of our baseline survey showed that all plots are largely independent from each other. The spatial autocorrelation of some of the variables was only significant in single short-or large-distance classes or with a small correlation coefficient. Further, the α- and β-diversity was low for all organism groups and the relationship between the biotic and abiotic baseline variables and the experimental treatments negligible. Overall, this suggests that the ecosystem variables are appropriate for future statistical analyses to clearly distinguish experimental effects from other effects and that the experimental site is representative for other oil-palm plantations, making results transferable.

Interestingly, we already see significant effects of the enrichment plantings on the bird and invertebrate fauna one year after the establishment of the experiment. We chose birds and invertebrates as study organisms, as they are used as bio-indicators to monitor changes in habitat quality. Previous studies have shown that ecosystem functioning is negatively affected by the loss in birds (Sekercioğlu, 2006; Tscharntke et al., 2008) and invertebrate diversity (Barnes et al., 2014; Ewers et al., 2015), highlighting their importance in ecosystems and, hence, their key role in conservation or restoration measures. Comparing the overall species numbers between 2013 and 2014, there were one bird species (5% loss) and 13 insect families (15% loss) in the LL less but a gain of 11 (12% gain) insect families in the HL. These differences may be due to annual fluctuations. In some cases, we see initial positive effects between the treatments (**Figures 5** and **6**).

The overall increase in bird richness on plots with trees compared to the control plots (**Figure 5A**) might be due to an overall increase in heterogeneity within the plantation; some of the planted trees (i.e., *Archidendron pauciflorum* and *Parkia speciosa*) had already reached considerable heights (>4 m) after the first year and provide habitat for nesting, roosting, and foraging (Thiollay, 1995), and might facilitate movement through the agricultural landscape (Harvey, 2000). This result supports findings that habitat heterogeneity and the presence of native trees are important factors determining bird diversity and composition (Sekercioğlu, 2002; Walther, 2002; Teuscher et al., 2015). At the plot scale, however, responses of birds were non-significant, indicating that overall habitat complexity at the plantation scale might be more important than at a local scale at this early stage of the experiment. More birds, especially frugivorous species, might be attracted by the tree islands when trees grow bigger and bring in fruits. Frugivorous birds were the second-most abundant feeding guild and the key role of birds as seed dispersers in tropical systems is well documented (Sekercioğlu, 2006; Whelan et al., 2008). This might positively affect succession and spontaneous colonization of plants in the near future (Cole et al., 2010).

Invertebrates responded to the enrichment plantings on a much smaller scale. There was an overall increase in the abundance of HL invertebrates on plots with trees across the whole plantation in year one compared to the control, but the abundance on plots with diversity level 0 was not significantly different from either. Furthermore, we see a positive relationship between the plot size and the difference in family richness of LL invertebrates and the difference in abundance of HL invertebrates, respectively, in year one compared to the baseline. These results suggest that tree planting alone had no significant effect on invertebrate communities. Only the combination of stop of fertilizer and pesticide application, changes in the light environment, the creation of new small-scale habitat structures through the planting of trees, and the cutting of oil palms might explain these positive responses of the invertebrate communities (see Tscharntke et al., 2011; Pywell et al., 2012). The increase in LL invertebrate family richness with increasing plot size may be correlated to increased litter input (Gillison et al., 2003) and increased stoichiometric diversity in the leaves (Ott et al., 2014). The significant positive relationship between invertebrate family richness as well as abundance and plot size suggests, however, that structural effects might be more important than tree diversity. We did not observe any shifts in the relative proportion of invertebrate biomass and abundance within trophic compartments between the baseline and year one and this might indicate a time-lag in the response of important ecosystem processes to differences in plant diversity, which was also reported from other studies (Cardinale et al., 2012; Eisenhauer et al., 2012; but see Schuldt et al., 2015). Invertebrates fulfill many tasks that are essential for ecosystem functioning including litter decomposition, predation, pollination, and herbivory. The design allows to disentangle the effects of plot size and tree diversity on the diversity and structure of different organism communities such as plants, birds, and invertebrates, and, herewith, to draw conclusions on changes in ecosystem functioning. The initial positive effects on birds and invertebrates, two organism group’s essential for the initiation of natural succession, are promising for further biodiversity enrichment in the future.

## Conclusion

EFForTS-BEE is designed to directly address questions about the potential of enriched oil-palm landscapes to maintain or enhance biodiversity and ecosystem functions and services whilst aiming to minimize economic losses. An expected outcome of the experiment is a combination of island size, tree diversity level, and composition that is above-average cost-effective and productive to achieve high gains in ecosystem functioning. This involves identifying the most well-performing tree species in their most productive composition under the conditions of an oil-palm plantation, which do not negatively affect oil palm yields. Initial positive responses of birds and invertebrates to the biodiversity enrichment treatments are promising and suggest that tree islands can be a suitable measure to enhance biodiversity in impoverished landscapes. The concept of planting tree islands in oil-palm landscapes might be similarly relevant for oil-palm estates managing large monoculture plantations as well as for smallholders seeking to diversify their production to reduce risks and their dependence on oil palm. In this context, the development of ‘Payment for Environmental Service’ schemes could help to make biodiversity enrichment more attractive for farmers. Depending on the goals of involved stakeholders, tree plantings could be adjusted to management forms such as agroforests or secondary forests for production of timber or conservation. Another possible application might be the re-transformation of existing illegal oil-palm plantations inside nature conservation areas into a more natural habitat. Nevertheless, the EFForTS-BEE does not satisfy the need for areas of ‘High Conservation Value’ which are an integral part of the designed plantation landscapes concept. In their function as source habitats, ‘High Conservation Value’ habitats are essential to recruit biota from and initiate successful natural succession in the EFForTS-BEE or other reduced-impact farming systems. Our long-term objectives are to provide basic knowledge on how to improve landscape connectivity with stepping stones to provide habitat for migrating biota and to buffer the inhospitality of oil-palm landscapes to enhance BEF at the landscape scale. With the results of the experiment, we aim at evaluating the effectiveness of enrichment plantings as part of designer plantation landscapes and at developing clear restoration instructions for oil palm farmers toward a more sustainable management of oil palm.

## Author Contributions

The experiment was designed by UB, YC, DH, HK, and MW. MT, ME, and AG planned, MT and AG carried out the establishment of the experimental plots with support from DB, LS, and BI. Data collection and analyses were done by AG and MT. The text was written by MT and AG with comments from UB, HK, YC, DH, MW, and ME.

## Conflict of Interest Statement

The authors declare that the research was conducted in the absence of any commercial or financial relationships that could be construed as a potential conflict of interest.

## Footnotes

1 www.uni-goettingen.de/crc990
