# Supplementary material for: Experimental Biodiversity Enrichment in Oil-Palm-Dominated Landscapes in Indonesia
Source: Front Plant Sci. 2016 Oct 17;7:1538. doi: 10.3389/fpls.2016.01538 (PMC5065973; doi:10.3389/fpls.2016.01538)
Supplement: Supplementary file 1 [file Presentation_1.PDF]

Appendix tables

Appendix Table 1: Plot infos

Plot ID	Latitude	Longitude	Plot size (m^2)	Tree diversity level	Number of fruit tree species	Number of timber tree species
1	-1.941619	103.251905	1600	1	1	0
2	-1.941743	103.252978	400	3	2	1
3	-1.943296	103.251765	400	2	1	1
4	-1.943206	103.253171	100	1	0	1
5	-1.944778	103.251792	1600	1	1	0
6	-1.944615	103.253150	25	1	0	1
7	-1.944689	103.255158	1600	3	1	2
8	-1.945868	103.249106	25	1	0	1
9	-1.945784	103.250588	100	3	2	1
10	-1.945945	103.251840	400	0	0	0
11	-1.945896	103.253220	100	1	0	1
12	-1.945888	103.254342	400	1	1	0
13	-1.945911	103.255925	100	1	0	1
14	-1.947283	103.249167	100	1	1	0
15	-1.947151	103.250424	400	1	1	0
16	-1.947333	103.251907	25	3	2	1
17	-1.947338	103.253148	400	1	0	1
18	-1.947168	103.254498	25	2	0	1
19	-1.947317	103.255865	400	6	3	3
20	-1.947337	103.257347	100	1	1	0
21	-1.948628	103.247800	100	6	3	3
22	-1.948734	103.249137	25	2	1	1
23	-1.948868	103.251317	1600	6	3	3
24	-1.948381	103.254313	1600	2	1	1
25	-1.948656	103.255886	25	1	1	0
26	-1.948487	103.257201	1600	2	0	1
27	-1.949921	103.246436	100	2	1	1
28	-1.950023	103.247777	25	1	1	0
29	-1.949964	103.248967	1600	3	2	1
30	-1.949809	103.252968	400	1	0	1
31	-1.949966	103.254488	25	1	1	0
32	-1.949976	103.255904	100	2	1	1
33	-1.950016	103.257276	400	3	1	2
34	-1.951426	103.245068	100	2	1	1
35	-1.951823	103.246590	1600	0	0	0
36	-1.951060	103.247721	400	2	1	1
37	-1.951176	103.248844	100	0	0	0
38	-1.951715	103.250417	400	1	0	1
39	-1.951383	103.251800	25	2	2	0
40	-1.951366	103.257142	25	0	0	0
41	-1.952674	103.243797	100	1	1	0
42	-1.952757	103.244984	25	1	0	1
43	-1.949631	103.258593	1600	1	1	0
44	-1.952709	103.247815	25	3	1	2
45	-1.953066	103.248695	1600	1	0	1
46	-1.954422	103.242421	1600	2	2	0
47	-1.953952	103.243710	400	2	1	1
48	-1.954103	103.245204	100	3	1	2
49	-1.953998	103.246627	1600	1	0	1
50	-1.954061	103.247820	25	6	3	3
51	-1.954249	103.249144	400	1	1	0
52	-1.955189	103.243481	1600	1	0	1

Appendix Tab. 2: Length-mass regression parameters for calculation of individual body masses from measured body lengths. For damaged individuals where body length could not be measured, body mass was substituted by species median body mass or order median body mass (for species with single individuals). 'Taxon' and 'Group' specify which animals the presented regression has been used for in this study. Regressions were available from the literature that estimate both dry and fresh mass ('Mass type') for different taxa. Appendix Tab. 4 presents the dry mass-fresh mass conversion, used to convert all estimated body masses to fresh mass. The equations and regression parameters, 'a' and 'b', are presented, as well as the size range the regressions were calculated from ('Min' and 'Max'). All regressions were taken from the literature ('Reference'), with different specific definitions of how body length was measured ('Details of body length measurement') and specificity of the given regression ('Regression specificity'). (Barnes et al. 2014; modified and extended)

Taxon	Group	Mass type	Equation M[mg], L[mm]	a	b	Min [mm]	Max [mm]	Reference	Details of body length measurement	Regression specificity
All insect taxa		Dry mass	M = a * (L)^b	0.035	2.173	0.9	17.6	Gruner (2003)	Tip of abdomen to end of head or carapace, excl. any appendages	Inferred, all insect taxa
All insect taxa		Dry mass	M = exp(a + b * log(L))	-3.628	2.494	2.13	54.51	Sample et al. (1993)	From frons to tip of abdomen excluding appendages	Inferred, all insect taxa
Araneae	Araneae < 2.5 Mm	Fresh mass	M = exp(a + b * log(L))	-1.958	2.746	0.56	2.5	Höfer and Ott (2009)	Edge of prosoma (without chelicerae) to edge of opisthosoma (excl spinnerets)	Group specific
Araneae	Hunting	Fresh mass	M = exp(a + b * log(L))	-2.108	3.017	0.67	36	Höfer and Ott (2009)	Edge of prosoma (without chelicerae) to edge of opisthosoma (excl spinnerets)	Group specific
Araneae	Web-Building	Fresh mass	M = exp(a + b * log(L))	-1.784	2.255	0.56	10.67	Höfer and Ott (2009)	Edge of prosoma (without chelicerae) to edge of opisthosoma (excl spinnerets)	Group specific
Araneae	Spiders Random	Fresh mass	M = exp(a + b * log(L))	-1.844	2.711	1.8	21.5	Edwards (1996)	Clypeus to tip of spinnerets	Group specific
Araneae	Anapidae	Fresh mass	M = exp(a + b * log(L))	-1.784	2.255	0.56	10.67	Höfer and Ott (2009)	Edge of prosoma (without chelicerae) to edge of opisthosoma (excl spinnerets)	Inferred, web-building
Araneae	Araneidae	Fresh mass	M = exp(a + b * log(L))	-1.923	2.923	2.10	21.20	Edwards (1996)	Clypeus to tip of spinnerets	Group specific
Araneae	Clubionidae	Fresh mass	M = exp(a + b * log(L))	-2.156	2.653	2.5	9	Edwards (1996)	Clypeus to tip of spinnerets	Group specific
Araneae	Corinnidae	Fresh mass	M = exp(a + b * log(L))	-2.108	3.017	0.67	36	Höfer and Ott (2009)	Edge of prosoma (without chelicerae) to edge of opisthosoma (excl spinnerets)	Inferred, hunting spiders
Araneae	Ctenidae	Fresh mass	M = exp(a + b * log(L))	-1.758	2.894	1.3	36	Höfer and Ott (2009)	Edge of prosoma (without chelicerae) to edge of opisthosoma (excl spinnerets)	Group specific
Araneae	Deinopidae	Fresh mass	M = exp(a + b * log(L))	-1.844	2.711	1.8	21.5	Edwards (1996)	Clypeus to tip of spinnerets	Inferred, spiders random sample
Araneae	Dysderidae	Fresh mass	M = exp(a + b * log(L))	-2.108	3.017	0.67	36	Höfer and Ott (2009)	Edge of prosoma (without chelicerae) to edge of opisthosoma (excl spinnerets)	Inferred, hunting spiders
Araneae	Gnaphosidae	Fresh mass	M = exp(a + b * log(L))	-2.83	3.055	3	13.1	Edwards (1996)	Clypeus to tip of spinnerets	Group specific

Araneae	Linyphiidae	Fresh mass	M = exp(a + b * log(L))	-1.892	2.754	1.5	5.5	Edwards (1996)	Clypeus to tip of spinnerets	Group specific
Araneae	Lycosidae	Fresh mass	M = exp(a + b * log(L))	-2.043	2.842	2	23.5	Edwards (1996)	Clypeus to tip of spinnerets	Group specific
Araneae	Miturgidae	Fresh mass	M = exp(a + b * log(L))	-2.83	3.055	3	13.1	Edwards (1996)	Clypeus to tip of spinnerets	Inferred, Gnaphosidae
Araneae	Mysmenidae	Fresh mass	M = exp(a + b * log(L))	-1.784	2.255	0.56	10.67	Höfer and Ott (2009)	Edge of prosoma (without chelicerae) to edge of opisthosoma (excl spinnerets)	Inferred, web-building
Araneae	Ochyroceratidae	Fresh mass	M = exp(a + b * log(L))	-1.784	2.255	0.56	10.67	Höfer and Ott (2009)	Edge of prosoma (without chelicerae) to edge of opisthosoma (excl spinnerets)	Inferred, web-building
Araneae	Oonopidae	Fresh mass	M = exp(a + b * log(L))	-2.039	2.666	0.67	2.5	Höfer and Ott (2009)	Edge of prosoma (without chelicerae) to edge of opisthosoma (excl spinnerets)	Group specific
Araneae	Oxyopidae	Fresh mass	M = exp(a + b * log(L))	-2.108	3.017	0.67	36	Höfer and Ott (2009)	Edge of prosoma (without chelicerae) to edge of opisthosoma (excl spinnerets)	Inferred, hunting spiders
Araneae	Philodromidae	Fresh mass	M = exp(a + b * log(L))	-1.985	2.940	2.50	8.60	Edwards (1996)	Clypeus to tip of spinnerets	Group specific
Araneae	Pholcidae	Fresh mass	M = exp(a + b * log(L))	-1.784	2.255	0.56	10.67	Höfer and Ott (2009)	Edge of prosoma (without chelicerae) to edge of opisthosoma (excl spinnerets)	Inferred, web-building
Araneau	Pisauridae	Fresh mass	M = exp(a + b * log(L))	-2.108	3.017	0.67	36	Höfer and Ott (2009)	Edge of prosoma (without chelicerae) to edge of opisthosoma (excl spinnerets)	Inferred, hunting spiders
Araneae	Salticidae	Fresh mass	M = exp(a + b * log(L))	-2.184	2.901	4.00	13.00	Edwards (1996)	Clypeus to tip of spinnerets	Group specific
Araneae	Sparassidae	Fresh mass	M = exp(a + b * log(L))	-2.108	3.017	0.67	36	Höfer and Ott (2009)	Edge of prosoma (without chelicerae) to edge of opisthosoma (excl spinnerets)	Inferred, hunting spiders
Araneae	Tetrablemmidae	Fresh mass	M = exp(a + b * log(L))	-2.039	2.666	0.67	2.5	Höfer and Ott (2009)	Edge of prosoma (without chelicerae) to edge of opisthosoma (excl spinnerets)	Inferred, Oonopidae
Araneae	Tetragnathidae	Fresh mass	M = exp(a + b * log(L))	-2.615	2.574	3.50	9.00	Edwards (1996)	Clypeus to tip of spinnerets	Group specific
Araneae	Theridiidae	Fresh mass	M = exp(a + b * log(L))	-1.577	2.907	1.50	7.50	Edwards (1996)	Clypeus to tip of spinnerets	Group specific
Araneae	Theridiosomatidae	Fresh mass	M = exp(a + b * log(L))	-1.784	2.255	0.56	10.67	Höfer and Ott (2009)	Edge of prosoma (without chelicerae) to edge of opisthosoma (excl spinnerets)	Inferred, web-building
Araneae	Thomisidae	Fresh mass	M = exp(a + b * log(L))	-1.644	2.973	1.80	8.00	Edwards (1996)	Clypeus to tip of spinnerets	Group specific
Araneae	Zodariidae	Fresh mass	M = exp(a + b * log(L))	-2.108	3.017	0.67	36	Höfer and Ott (2009)	Edge of prosoma (without chelicerae) to edge of opisthosoma (excl spinnerets)	Inferred, hunting spiders
Blattodea	Blaberidae	Dry mass	M = exp(a + b * log(L))	-3.980	2.760	2.20	14.00	Wardhaugh (2013)	Front of labrum to tip of abdomen (excl. cerci or ovipositors) or tip of elytra (longest)	Inferred, Blattodea
Blattodea	Blattellidae	Dry mass	M = exp(a + b * log(L))	-3.980	2.760	2.20	14.00	Wardhaugh (2013)	Front of labrum to tip of abdomen (excl. cerci or ovipositors) or tip of elytra (longest)	Inferred, Blattodea
Blattodea	Blattidae	Dry mass	M = exp(a + b * log(L))	-3.980	2.760	2.20	14.00	Wardhaugh (2013)	Front of labrum to tip of abdomen (excl. cerci or ovipositors) or tip of elytra (longest)	Inferred, Blattodea

Coleoptera	Aderidae	Dry mass	M = exp(a + b * log(L))	-3.247	2.492	3.34	34.82	Sample et al. (1993)	From frons to tip of abdomen excluding appendages	Inferred, Coleoptera
Coleoptera	Anthicidae	Dry mass	M = exp(a + b * log(L))	-3.247	2.492	3.34	34.82	Sample et al. (1993)	From frons to tip of abdomen excluding appendages	Inferred, Coleoptera
Coleoptera	Anthribidae	Dry mass	M = exp(a + b * log(L))	-3.247	2.492	3.34	34.82	Sample et al. (1993)	From frons to tip of abdomen excluding appendages	Inferred, Coleoptera
Coleoptera	Carabidae	Dry mass	M = a * L^b	0.0237	2.7054	2.88	24	Lang et al. (1997)	Measured from anterior tip of head to posterior of abdomen excluding any appendages	Group specific
Coleoptera	Chrysomelidae	Dry mass	M = exp(a + b * log(L))	-2.427	2.171	3.34	7.84	Sample et al. (1993)	From frons to tip of abdomen excluding appendages	Group specific
Coleoptera	Corylophidae	Dry mass	M = exp(a + b * log(L))	-3.247	2.492	3.34	34.82	Sample et al. (1993)	From frons to tip of abdomen excluding appendages	Inferred, Coleoptera
Coleoptera	Colydiidae	Dry mass	M = exp(a + b * log(L))	-3.247	2.492	3.34	34.82	Sample et al. (1993)	From frons to tip of abdomen excluding appendages	Inferred, Coleoptera
Coleoptera	Discolomidae	Dry mass	M = exp(a + b * log(L))	-3.247	2.492	3.34	34.82	Sample et al. (1993)	From frons to tip of abdomen excluding appendages	Inferred, Coleoptera
Coleoptera	Hydraenidae	Dry mass	M = exp(a + b * log(L))	-3.247	2.492	3.34	34.82	Sample et al. (1993)	From frons to tip of abdomen excluding appendages	Inferred, Coleoptera
Coleoptera	Lagrioidinae	Dry mass	M = exp(a + b * log(L))	-3.247	2.492	3.34	34.82	Sample et al. (1993)	From frons to tip of abdomen excluding appendages	Inferred, Coleoptera
Coleoptera	Pselaphidae	Dry mass	M = exp(a + b * log(L))	-3.247	2.492	3.34	34.82	Sample et al. (1993)	From frons to tip of abdomen excluding appendages	Inferred, Coleoptera
Coleoptera	Scolytidae	Dry mass	M = exp(a + b * log(L))	-3.247	2.492	3.34	34.82	Sample et al. (1993)	From frons to tip of abdomen excluding appendages	Inferred, Coleoptera
Coleoptera	Curculionidae	Dry mass	M = exp(a + b * log(L))	-3.247	2.492	3.34	34.82	Sample et al. (1993)	From frons to tip of abdomen excluding appendages	Inferred, Coleoptera
Coleoptera	Elateridae	Dry mass	M = a * L^b	0.0138	2.595	1.65	10.3	Gruner (2003)	Tip of abdomen to end of head or carapace, excl. any appendages	Inferred, slender beetles
Coleoptera	Nitidulidae	Dry mass	M = exp(a + b * log(L))	-3.247	2.492	3.34	34.82	Sample et al. (1993)	From frons to tip of abdomen excluding appendages	Inferred, Coleoptera
Coleoptera	Phalacridae	Dry mass	M = exp(a + b * log(L))	-3.247	2.492	3.34	34.82	Sample et al. (1993)	From frons to tip of abdomen excluding appendages	Inferred, Coleoptera
Coleoptera	Ptiliidae	Dry mass	M = exp(a + b * log(L))	-3.247	2.492	3.34	34.82	Sample et al. (1993)	From frons to tip of abdomen excluding appendages	Inferred, Coleoptera
Coleoptera	Scarabaeidae	Dry mass	M = exp(a + b * log(L))	-2.448	2.494	4.24	24.79	Sample et al. (1993)	From frons to tip of abdomen excluding appendages	Group specific
Coleoptera	Scydmaenidae	Dry mass	M = exp(a + b * log(L))	-3.247	2.492	3.34	34.82	Sample et al. (1993)	From frons to tip of abdomen excluding appendages	Inferred, Coleoptera
Coleoptera	Silvanidae	Dry mass	M = a * L^b	0.0138	2.595	1.65	10.3	Gruner (2003)	Tip of abdomen to end of head or carapace, excl. any appendages	Inferred, slender beetles
Coleoptera	Staphylinidae	Dry mass	M = a * L^b	0.0134	2.26	2.2	13.6	Lang et al. (1997)	Measured from anterior tip of head to posterior of abdomen excluding any appendages	Group specific

Coleoptera	Tenebrionidae	Dry mass	M = exp(a + b * log(L))	-0.043	1.2	5.65	13.39	Sample et al. (1993)	From frons to tip of abdomen excluding appendages	Group specific
Dermaptera	Anisolabididae	Dry mass	M = exp(a + b * log(L))	-3.628	2.494	2.13	54.51	Sample et al. (1993)	From frons to tip of abdomen excluding appendages	Inferred, all insect taxa
Dermaptera	Labiduridae	Dry mass	M = exp(a + b * log(L))	-3.628	2.494	2.13	54.51	Sample et al. (1993)	From frons to tip of abdomen excluding appendages	Inferred, all insect taxa
Dermaptera	Labiidae	Dry mass	M = exp(a + b * log(L))	-3.628	2.494	2.13	54.51	Sample et al. (1993)	From frons to tip of abdomen excluding appendages	Inferred, all insect taxa
Dermaptera	Spongiphoridae	Dry mass	M = exp(a + b * log(L))	-3.628	2.494	2.13	54.51	Sample et al. (1993)	From frons to tip of abdomen excluding appendages	Inferred, all insect taxa
Dermaptera	Uniden	Dry mass	M = exp(a + b * log(L))	-3.628	2.494	2.13	54.51	Sample et al. (1993)	From frons to tip of abdomen excluding appendages	Inferred, all insect taxa
Diplura	Campodeidae	Dry mass	M = a * (L)^b	0.034	2.191	0.9	17.6	Gruner (2003)	Tip of abdomen to end of head or carapace, excl. any appendages	Inferred, general arthropod
Diplura	Japygidae	Dry mass	M = a * (L)^b	0.034	2.191	0.9	17.6	Gruner (2003)	Tip of abdomen to end of head or carapace, excl. any appendages	Inferred, general arthropod
Diplura	Stratiopmydae	Dry mass	M = a * (L)^b	0.034	2.191	0.9	17.6	Gruner (2003)	Tip of abdomen to end of head or carapace, excl. any appendages	Inferred, general arthropod
Diplura	Uniden	Dry mass	M = a * (L)^b	0.034	2.191	0.9	17.6	Gruner (2003)	Tip of abdomen to end of head or carapace, excl. any appendages	Inferred, general arthropod
Diptera	Adults	Dry mass	M = a * (L)^b	0.0153	2.573	1.75	8.6	Gruner (2003)	Tip of abdomen to end of head or carapace, excl. any appendages	Original, Diptera adult
Diptera	Asteiidae	Dry mass	M = a * (L)^b	0.0153	2.573	1.75	8.6	Gruner (2003)	Tip of abdomen to end of head or carapace, excl. any appendages	Original, Diptera adult
Diptera	Bibionidae	Dry mass	M = a * (L)^b	0.035	2.173	0.9	17.6	Gruner (2003)	Tip of abdomen to end of head or carapace, excl. any appendages	Inferred, all insect taxa
Diptera	Calliphoridae	Dry mass	M = a * (L)^b	0.0153	2.573	1.75	8.6	Gruner (2003)	Tip of abdomen to end of head or carapace, excl. any appendages	Original, Diptera adult
Diptera	Cecidomyiidae	Dry mass	M = a * (L)^b	0.035	2.173	0.9	17.6	Gruner (2003)	Tip of abdomen to end of head or carapace, excl. any appendages	Inferred, all insect taxa
Diptera	Ceratopogonidae	Dry mass	M = a * (L)^b	0.0153	2.573	1.75	8.6	Gruner (2003)	Tip of abdomen to end of head or carapace, excl. any appendages	Original, Diptera adult
Diptera	Chironomidae	Dry mass	M = a * (L)^b	0.0153	2.573	1.75	8.6	Gruner (2003)	Tip of abdomen to end of head or carapace, excl. any appendages	Original, Diptera adult
Diptera	Chloropidae	Dry mass	M = a * (L)^b	0.0153	2.573	1.75	8.6	Gruner (2003)	Tip of abdomen to end of head or carapace, excl. any appendages	Original, Diptera adult
Diptera	Clusiidae	Dry mass	M = a * (L)^b	0.0153	2.573	1.75	8.6	Gruner (2003)	Tip of abdomen to end of head or carapace, excl. any appendages	Original, Diptera adult
Diptera	Culicidae	Dry mass	M = a * (L)^b	0.0153	2.573	1.75	8.6	Gruner (2003)	Tip of abdomen to end of head or carapace, excl. any appendages	Original, Diptera adult
Diptera	Deuterophlebiidae	Dry mass	M = a * (L)^b	0.0153	2.573	1.75	8.6	Gruner (2003)	Tip of abdomen to end of head or carapace, excl. any appendages	Original, Diptera adult

Diptera	Dolichopodidae	Dry mass	M = a * (L)^b	0.0153	2.573	1.75	8.6	Gruner (2003)	Tip of abdomen to end of head or carapace, excl. any appendages	Original, Diptera adult
Diptera	Drosophilidae	Dry mass	M = a * (L)^b	0.0153	2.573	1.75	8.6	Gruner (2003)	Tip of abdomen to end of head or carapace, excl. any appendages	Original, Diptera adult
Diptera	Lauxaniidae	Dry mass	M = a * (L)^b	0.0153	2.573	1.75	8.6	Gruner (2003)	Tip of abdomen to end of head or carapace, excl. any appendages	Original, Diptera adult
Diptera	Muscidae	Dry mass	M = a * (L)^b	0.0153	2.573	1.75	8.6	Gruner (2003)	Tip of abdomen to end of head or carapace, excl. any appendages	Original, Diptera adult
Diptera	Mycetophylidae	Dry mass	M = a * (L)^b	0.0153	2.573	1.75	8.6	Gruner (2003)	Tip of abdomen to end of head or carapace, excl. any appendages	Original, Diptera adult
Diptera	Opomyzidae	Dry mass	M = a * (L)^b	0.0153	2.573	1.75	8.6	Gruner (2003)	Tip of abdomen to end of head or carapace, excl. any appendages	Original, Diptera adult
Diptera	Phoridae	Dry mass	M = a * (L)^b	0.0153	2.573	1.75	8.6	Gruner (2003)	Tip of abdomen to end of head or carapace, excl. any appendages	Original, Diptera adult
Diptera	Piophilidae	Dry mass	M = a * (L)^b	0.0153	2.573	1.75	8.6	Gruner (2003)	Tip of abdomen to end of head or carapace, excl. any appendages	Original, Diptera adult
Diptera	Psychodidae	Dry mass	M = a * (L)^b	0.0153	2.573	1.75	8.6	Gruner (2003)	Tip of abdomen to end of head or carapace, excl. any appendages	Original, Diptera adult
Diptera	Sciaridae	Dry mass	M = a * (L)^b	0.0153	2.573	1.75	8.6	Gruner (2003)	Tip of abdomen to end of head or carapace, excl. any appendages	Original, Diptera adult
Diptera	Simuliidae	Dry mass	M = a * (L)^b	0.0153	2.573	1.75	8.6	Gruner (2003)	Tip of abdomen to end of head or carapace, excl. any appendages	Original, Diptera adult
Diptera	Sphaeroceridae	Dry mass	M = a * (L)^b	0.0153	2.573	1.75	8.6	Gruner (2003)	Tip of abdomen to end of head or carapace, excl. any appendages	Original, Diptera adult
Diptera	Pipunculidae	Dry mass	M = a * (L)^b	0.0153	2.573	1.75	8.6	Gruner (2003)	Tip of abdomen to end of head or carapace, excl. any appendages	Original, Diptera adult
Diptera	Scatopsidae	Dry mass	M = a * (L)^b	0.0153	2.573	1.75	8.6	Gruner (2003)	Tip of abdomen to end of head or carapace, excl. any appendages	Original, Diptera adult
Diptera	Tipulidae	Dry mass	M = a * (L)^b	0.0153	2.573	1.75	8.6	Gruner (2003)	Tip of abdomen to end of head or carapace, excl. any appendages	Original, Diptera adult
Diptera	Uniden	Dry mass	M = a * (L)^b	0.0153	2.573	1.75	8.6	Gruner (2003)	Tip of abdomen to end of head or carapace, excl. any appendages	Original, Diptera adult
Ephemeroptera	Baetidae	Dry mass	M = a * (L)^b	0.035	2.173	0.9	17.6	Gruner (2003)	Tip of abdomen to end of head or carapace, excl. any appendages	Inferred, all insect taxa
Chilopoda	All	Dry mass	M = exp(a + b * log(L))	-4.049	2.18	4	47	Gowing and Recher (1984)	Not mentioned	Inferred, Chilopoda
Geophilomorpha	Dignathodontidae	Dry mass	M = exp(a + b * log(L))	-4.049	2.18	4	47	Gowing and Recher (1984)	Not mentioned	Inferred, Chilopoda
Geophilomorpha	Geophilidae	Dry mass	M = exp(a + b * log(L))	-4.049	2.18	4	47	Gowing and Recher (1984)	Not mentioned	Inferred, Chilopoda
Geophilomorpha	Mecistocephalidae	Dry mass	M = exp(a + b * log(L))	-4.049	2.18	4	47	Gowing and Recher (1984)	Not mentioned	Inferred, Chilopoda

Diplopoda	All	Dry mass	M = exp(a + b * log(L))	-4.591	2.543	11.0	47.0	Gowing and Recher (1984)	Not mentioned	Inferred, Diplopoda
Glomerida	Glomeridae	Dry mass	M = exp(a + b * log(L))	-4.591	2.543	11.0	47.0	Gowing and Recher (1984)	Not mentioned	Inferred, Diplopoda
Hemiptera	Aleyrodidae	Dry mass	M = exp(a + b * log(L))	-2.823	2.225	2.13	13.25	Sample et al. (1993)	From frons to tip of abdomen excluding appendages	Inferred, homoptera
Hemiptera	Alydidae	Dry mass	M = exp(a + b * log(L))	-4.784	3.075	3.2	40.23	Sample et al. (1993)	From frons to tip of abdomen excluding appendages	Original, Hemiptera
Hemiptera	Anthocoridae	Dry mass	M = exp(a + b * log(L))	-4.784	3.075	3.2	40.23	Sample et al. (1993)	From frons to tip of abdomen excluding appendages	Original, Hemiptera
Hemiptera	Aphididae	Dry mass	M = exp(a + b * log(L))	-2.823	2.225	2.13	13.25	Sample et al. (1993)	From frons to tip of abdomen excluding appendages	Inferred, homoptera
Hemiptera	Aradidae	Dry mass	M = exp(a + b * log(L))	-4.784	3.075	3.2	40.23	Sample et al. (1993)	From frons to tip of abdomen excluding appendages	Original, Hemiptera
Hemiptera	Ceratocombidae	Dry mass	M = exp(a + b * log(L))	-4.784	3.075	3.2	40.23	Sample et al. (1993)	From frons to tip of abdomen excluding appendages	Original, Hemiptera
Hemiptera	Cicadellidae	Dry mass	M = exp(a + b * log(L))	-3.735	2.561	2.13	13.25	Sample et al. (1993)	From frons to tip of abdomen excluding appendages	Group specific
Hemiptera	Cixiidae	Dry mass	M = exp(a + b * log(L))	-4.784	3.075	3.2	40.23	Sample et al. (1993)	From frons to tip of abdomen excluding appendages	Inferred, Hemiptera
Hemiptera	Coreidae	Dry mass	M = exp(a + b * log(L))	-4.784	3.075	3.2	40.23	Sample et al. (1993)	From frons to tip of abdomen excluding appendages	Inferred, Hemiptera
Hemiptera	Cydnidae	Dry mass	M = exp(a + b * log(L))	-4.784	3.075	3.2	40.23	Sample et al. (1993)	From frons to tip of abdomen excluding appendages	Inferred, Hemiptera
Hemiptera	Delphacidae	Dry mass	M = exp(a + b * log(L))	-2.823	2.225	2.13	13.25	Sample et al. (1993)	From frons to tip of abdomen excluding appendages	Inferred, homoptera
Hemiptera	Derbidae	Dry mass	M = exp(a + b * log(L))	-2.823	2.225	2.13	13.25	Sample et al. (1993)	From frons to tip of abdomen excluding appendages	Inferred, homoptera
Hemiptera	Dictyopharidae	Dry mass	M = exp(a + b * log(L))	-2.823	2.225	2.13	13.25	Sample et al. (1993)	From frons to tip of abdomen excluding appendages	Inferred, homoptera
Hemiptera	Dipsocoridae	Dry mass	M = exp(a + b * log(L))	-4.784	3.075	3.2	40.23	Sample et al. (1993)	From frons to tip of abdomen excluding appendages	Inferred, Hemiptera
Hemiptera	Eriosomatidae	Dry mass	M = exp(a + b * log(L))	-2.823	2.225	2.13	13.25	Sample et al. (1993)	From frons to tip of abdomen excluding appendages	Inferred, homoptera
Hemiptera	Flatidae	Dry mass	M = exp(a + b * log(L))	-2.823	2.225	2.13	13.25	Sample et al. (1993)	From frons to tip of abdomen excluding appendages	Inferred, homoptera
Hemiptera	Geocoridae	Dry mass	M = exp(a + b * log(L))	-4.784	3.075	3.2	40.23	Sample et al. (1993)	From frons to tip of abdomen excluding appendages	Inferred, Hemiptera
Hemiptera	Hydrometridae	Dry mass	M = exp(a + b * log(L))	-4.784	3.075	3.2	40.23	Sample et al. (1993)	From frons to tip of abdomen excluding appendages	Inferred, Hemiptera
Hemiptera	Largidae	Dry mass	M = exp(a + b * log(L))	-4.784	3.075	3.2	40.23	Sample et al. (1993)	From frons to tip of abdomen excluding appendages	Inferred, Hemiptera

Hemiptera	Lophopidae	Dry mass	M = exp(a + b * log(L))	-2.823	2.225	2.13	13.25	Sample et al. (1993)	From frons to tip of abdomen excluding appendages	Inferred, homoptera
Hemiptera	Lygaeidae	Dry mass	M = exp(a + b * log(L))	-4.784	3.075	3.2	40.23	Sample et al. (1993)	From frons to tip of abdomen excluding appendages	Inferred, Hemiptera
Hemiptera	Meenoplidae	Dry mass	M = exp(a + b * log(L))	-2.823	2.225	2.13	13.25	Sample et al. (1993)	From frons to tip of abdomen excluding appendages	Inferred, homoptera
Hemiptera	Miridae	Dry mass	M = exp(a + b * log(L))	-4.784	3.075	3.2	40.23	Sample et al. (1993)	From frons to tip of abdomen excluding appendages	Inferred, Hemiptera
Hemiptera	Nabidae	Dry mass	M = exp(a + b * log(L))	-4.784	3.075	3.2	40.23	Sample et al. (1993)	From frons to tip of abdomen excluding appendages	Inferred, Hemiptera
Hemiptera	Pentatomidae	Dry mass	M = exp(a + b * log(L))	-4.197	3.053	6.35	16.73	Sample et al. (1993)	From frons to tip of abdomen excluding appendages	Group specific
Hemiptera	Pseudococcidae	Dry mass	M = exp(a + b * log(L))	-2.823	2.225	2.13	13.25	Sample et al. (1993)	From frons to tip of abdomen excluding appendages	Inferred, homoptera
Hemiptera	Reduviidae	Dry mass	M = exp(a + b * log(L))	-4.784	3.075	3.2	40.23	Sample et al. (1993)	From frons to tip of abdomen excluding appendages	Inferred, Hemiptera
Hemiptera	Ricaniidae	Dry mass	M = exp(a + b * log(L))	-2.823	2.225	2.13	13.25	Sample et al. (1993)	From frons to tip of abdomen excluding appendages	Inferred, homoptera
Hemiptera	Schizopteridae	Dry mass	M = exp(a + b * log(L))	-4.784	3.075	3.2	40.23	Sample et al. (1993)	From frons to tip of abdomen excluding appendages	Inferred, Hemiptera
Hemiptera	Tingidae	Dry mass	M = exp(a + b * log(L))	-4.784	3.075	3.2	40.23	Sample et al. (1993)	From frons to tip of abdomen excluding appendages	Inferred, Hemiptera
Hemiptera	Tropiduchidae	Dry mass	M = exp(a + b * log(L))	-2.823	2.225	2.13	13.25	Sample et al. (1993)	From frons to tip of abdomen excluding appendages	Inferred, homoptera
Hemiptera	Uniden	Dry mass	M = exp(a + b * log(L))	-4.784	3.075	3.2	40.23	Sample et al. (1993)	From frons to tip of abdomen excluding appendages	Inferred, Hemiptera
Hymenoptera	Aphelinidae	Dry mass	M = exp(a + b * log(L))	-3.336	2.104	1	12	Gowing and Recher (1984)	Not mentioned	Inferred, Hym. Excl Formicidae
Hymenoptera	Bethylidae	Dry mass	M = exp(a + b * log(L))	-3.336	2.104	1	12	Gowing and Recher (1984)	Not mentioned	Inferred, Hym. Excl Formicidae
Hymenoptera	Braconidae	Dry mass	M = exp(a + b * log(L))	-3.336	2.104	1	12	Gowing and Recher (1984)	Not mentioned	Inferred, Hym. Excl Formicidae
Hymenoptera	Ceraphronidae	Dry mass	M = exp(a + b * log(L))	-3.336	2.104	1	12	Gowing and Recher (1984)	Not mentioned	Inferred, Hym. Excl Formicidae
Hymenoptera	Chalcididae	Dry mass	M = exp(a + b * log(L))	-3.336	2.104	1	12	Gowing and Recher (1984)	Not mentioned	Inferred, Hym. Excl Formicidae
Hymenoptera	Cynipidae	Dry mass	M = exp(a + b * log(L))	-3.336	2.104	1	12	Gowing and Recher (1984)	Not mentioned	Inferred, Hym. Excl Formicidae
Hymenoptera	Diapriidae	Dry mass	M = exp(a + b * log(L))	-3.336	2.104	1	12	Gowing and Recher (1984)	Not mentioned	Inferred, Hym. Excl Formicidae
Hymenoptera	Dryinidae	Dry mass	M = exp(a + b * log(L))	-3.336	2.104	1	12	Gowing and Recher (1984)	Not mentioned	Inferred, Hym. Excl Formicidae

Hymenoptera	Dryniidae	Dry mass	M = exp(a + b * log(L))	-3.336	2.104	1	12	Gowing and Recher (1984)	Not mentioned	Inferred, Hym. Excl Formicidae
Hymenoptera	Encyrtidae	Dry mass	M = exp(a + b * log(L))	-3.336	2.104	1	12	Gowing and Recher (1984)	Not mentioned	Inferred, Hym. Excl Formicidae
Hymenoptera	Eucoilidae	Dry mass	M = exp(a + b * log(L))	-3.336	2.104	1	12	Gowing and Recher (1984)	Not mentioned	Inferred, Hym. Excl Formicidae
Hymenoptera	Eulophidae	Dry mass	M = exp(a + b * log(L))	-3.336	2.104	1	12	Gowing and Recher (1984)	Not mentioned	Inferred, Hym. Excl Formicidae
Hymenoptera	Figitidae	Dry mass	M = exp(a + b * log(L))	-3.336	2.104	1	12	Gowing and Recher (1984)	Not mentioned	Inferred, Hym. Excl Formicidae
Hymenoptera	Formicidae	Dry mass	M = exp(a + b * log(L))	-3.996	2.489	2	18	Gowing and Recher (1984)	Not mentioned	Group specific
Hymenoptera	Ichneumonidae	Dry mass	M = exp(a + b * log(L))	-3.336	2.104	1	12	Gowing and Recher (1984)	Not mentioned	Inferred, Hym. Excl Formicidae
Hymenoptera	Mymaridae	Dry mass	M = exp(a + b * log(L))	-3.336	2.104	1	12	Gowing and Recher (1984)	Not mentioned	Inferred, Hym. Excl Formicidae
Hymenoptera	Mymarommatidae	Dry mass	M = exp(a + b * log(L))	-3.336	2.104	1	12	Gowing and Recher (1984)	Not mentioned	Inferred, Hym. Excl Formicidae
Hymenoptera	Platygastridae	Dry mass	M = exp(a + b * log(L))	-3.336	2.104	1	12	Gowing and Recher (1984)	Not mentioned	Inferred, Hym. Excl Formicidae
Hymenoptera	Scelionidae	Dry mass	M = exp(a + b * log(L))	-3.336	2.104	1	12	Gowing and Recher (1984)	Not mentioned	Inferred, Hym. Excl Formicidae
Hymenoptera	Sphecidae	Dry mass	M = exp(a + b * log(L))	-3.336	2.104	1	12	Gowing and Recher (1984)	Not mentioned	Inferred, Hym. Excl Formicidae
Hymenoptera	Tiphiidae	Dry mass	M = exp(a + b * log(L))	-3.336	2.104	1	12	Gowing and Recher (1984)	Not mentioned	Inferred, Hym. Excl Formicidae
Hymenoptera	Trichogrammatidae	Dry mass	M = exp(a + b * log(L))	-3.336	2.104	1	12	Gowing and Recher (1984)	Not mentioned	Inferred, Hym. Excl Formicidae
Hymenoptera	Vespidae	Dry mass	M = exp(a + b * log(L))	-3.336	2.104	1	12	Gowing and Recher (1984)	Not mentioned	Inferred, Hym. Excl Formicidae
Isopoda	All	Dry mass	M = exp(a + b * log(L))	-4.81	3.44	2.7	8	Wardhaugh (2013)	Front of labrum to tip of abdomen (excl. cerci or ovipositors) or tip of elytra (longest)	Original, Isopoda
Isopoda	Oniscidae	Dry mass	M = exp(a + b * log(L))	-4.81	3.44	2.7	8	Wardhaugh (2013)	Front of labrum to tip of abdomen (excl. cerci or ovipositors) or tip of elytra (longest)	Original, Isopoda
Isopoda	Philosciidae	Dry mass	M = exp(a + b * log(L))	-4.81	3.44	2.7	8	Wardhaugh (2013)	Front of labrum to tip of abdomen (excl. cerci or ovipositors) or tip of elytra (longest)	Original, Isopoda
Isopoda	Armadillidae	Dry mass	M = exp(a + b * log(L))	-4.81	3.44	2.7	8	Wardhaugh (2013)	Front of labrum to tip of abdomen (excl. cerci or ovipositors) or tip of elytra (longest)	Original, Isopoda
Isopoda	Termitidae	Dry mass	M = exp(a + b * log(L))	-4.81	3.44	2.7	8	Wardhaugh (2013)	Front of labrum to tip of abdomen (excl. cerci or ovipositors) or tip of elytra	Original, Isopoda

									(longest)	
Isoptera	Rhinotermitidae	Dry mass	M = e^a * L^b	-5.802	3.177	3.30	5.60	Johnson and Strong (2000)	Head to end of abdomen	Inferred, Isoptera
Isoptera	Termitidae	Dry mass	M = e^a * L^b	-5.802	3.177	3.30	5.60	Johnson and Strong (2000)	Head to end of abdomen	Inferred, Isoptera
Lepidoptera	Acanthopteroctetidae	Dry mass	M = exp(a + b * log(L))	-5.036	3.122	2.76	40.73	Sample et al. (1993)	Frons to tip of abdomen (excl. antennae, ovipositors, wings etc.)	Inferred, Lepidoptera
Lepidoptera	Acanthopteroctetidae	Dry mass	M = exp(a + b * log(L))	-5.036	3.122	2.76	40.73	Sample et al. (1993)	Frons to tip of abdomen (excl. antennae, ovipositors, wings etc.)	Inferred, Lepidoptera
Lepidoptera	Gelechiidae	Dry mass	M = exp(a + b * log(L))	-5.036	3.122	2.76	40.73	Sample et al. (1993)	Frons to tip of abdomen (excl. antennae, ovipositors, wings etc.)	Inferred, Lepidoptera
Lepidoptera	Geometridae	Dry mass	M = exp(a + b * log(L))	-5.036	3.122	2.76	40.73	Sample et al. (1993)	Frons to tip of abdomen (excl. antennae, ovipositors, wings etc.)	Inferred, Lepidoptera
Lepidoptera	Gracillariidae	Dry mass	M = exp(a + b * log(L))	-5.036	3.122	2.76	40.73	Sample et al. (1993)	Frons to tip of abdomen (excl. antennae, ovipositors, wings etc.)	Inferred, Lepidoptera
Lepidoptera	Noctuidae	Dry mass	M = exp(a + b * log(L))	-5.036	3.122	2.76	40.73	Sample et al. (1993)	Frons to tip of abdomen (excl. antennae, ovipositors, wings etc.)	Inferred, Lepidoptera
Lepidoptera	Pyralidae	Dry mass	M = exp(a + b * log(L))	-5.036	3.122	2.76	40.73	Sample et al. (1993)	Frons to tip of abdomen (excl. antennae, ovipositors, wings etc.)	Inferred, Lepidoptera
Lepidoptera	Thyrididae	Dry mass	M = exp(a + b * log(L))	-5.036	3.122	2.76	40.73	Sample et al. (1993)	Frons to tip of abdomen (excl. antennae, ovipositors, wings etc.)	Inferred, Lepidoptera
Lepidoptera	Tineidae	Dry mass	M = exp(a + b * log(L))	-5.036	3.122	2.76	40.73	Sample et al. (1993)	Frons to tip of abdomen (excl. antennae, ovipositors, wings etc.)	Inferred, Lepidoptera
Lepidoptera	Tortricidae	Dry mass	M = exp(a + b * log(L))	-5.036	3.122	2.76	40.73	Sample et al. (1993)	Frons to tip of abdomen (excl. antennae, ovipositors, wings etc.)	Inferred, Lepidoptera
Lepidoptera	Zygaenidae	Dry mass	M = exp(a + b * log(L))	-5.036	3.122	2.76	40.73	Sample et al. (1993)	Frons to tip of abdomen (excl. antennae, ovipositors, wings etc.)	Inferred, Lepidoptera
Lepidoptera	Uniden	Dry mass	M = exp(a + b * log(L))	-5.036	3.122	2.76	40.73	Sample et al. (1993)	Frons to tip of abdomen (excl. antennae, ovipositors, wings etc.)	Inferred, Lepidoptera
Lithobiomorpha	Henicopidae	Dry mass	M = exp(a + b * log(L))	-4.049	2.18	4	47	Gowing and Recher (1984)	Not mentioned	Inferred, Chilopoda
Mantodea	Mantidae	Dry mass	M = exp(a + b * log(L))	-6.340	3.010	6.00	66.00	Wardhaugh (2013)	Front of labrum to tip of abdomen (excl. cerci or ovipositors) or tip of elytra (longest)	Group specific
Neuroptera	Chrysopidae	Dry mass	M = exp(a + b * log(L))	-4.483	2.570	3.45	54.51	Sample et al. (1993)	Frons to tip of abdomen (excl. antennae, ovipositors, wings etc.)	Inferred, Neuroptera
Opiliones	All	Fresh mass	M = exp(a + b * log(L))	-0.899	2.984	0.57	6.9	Höfer and Ott (2009)	Edge of prosoma (without chelicerae) to edge of opisthosoma (excl spinnerets)	Inferred, Opiliones
Orthoptera	Acrididae	Dry mass	M = exp(a + b * log(L))	-3.17	2.61	2.3	33	Wardhaugh (2013)	Front of labrum to tip of abdomen (excl. cerci or ovipositors) or tip of elytra (longest)	Inferred, Orthoptera
Orthoptera	Gryllidae	Dry mass	M = exp(a + b * log(L))	-3.17	2.61	2.3	33	Wardhaugh (2013)	Front of labrum to tip of abdomen (excl. cerci or ovipositors) or tip of elytra (longest)	Inferred, Orthoptera
Orthoptera	Tetrigidae	Dry mass	M = exp(a + b * log(L))	-3.17	2.61	2.3	33	Wardhaugh (2013)	Front of labrum to tip of abdomen (excl.	Inferred, Orthoptera

									cerci or ovipositors) or tip of elytra (longest)	
Orthoptera	Gryllacrididae	Dry mass	M = exp(a + b * log(L))	-3.17	2.61	2.3	33	Wardhaugh (2013)	Front of labrum to tip of abdomen (excl. cerci or ovipositors) or tip of elytra (longest)	Inferred, Orthoptera
Orthoptera	Tettigoniidae	Dry mass	M = exp(a + b * log(L))	-3.17	2.61	2.3	33	Wardhaugh (2013)	Front of labrum to tip of abdomen (excl. cerci or ovipositors) or tip of elytra (longest)	Inferred, Orthoptera
Orthoptera	Tridactylidae	Dry mass	M = exp(a + b * log(L))	-3.17	2.61	2.3	33	Wardhaugh (2013)	Front of labrum to tip of abdomen (excl. cerci or ovipositors) or tip of elytra (longest)	Inferred, Orthoptera
Polydesmida	Paradoxosomatidae	Dry mass	M = exp(a + b * log(L))	-4.591	2.543	11.0	47.0	Gowing and Recher (1984)	Not mentioned	Inferred, Diplopoda
Polydesmida	Platyrhacidae	Dry mass	M = exp(a + b * log(L))	-4.591	2.543	11.0	47.0	Gowing and Recher (1984)	Not mentioned	Inferred, Diplopoda
Polydesmida	Cryptodesmidae	Dry mass	M = exp(a + b * log(L))	-4.591	2.543	11.0	47.0	Gowing and Recher (1984)	Not mentioned	Inferred, Diplopoda
Polydesmida	Dalodesmidae	Dry mass	M = exp(a + b * log(L))	-4.591	2.543	11.0	47.0	Gowing and Recher (1984)	Not mentioned	Inferred, Diplopoda
Polydesmida	Haplodesmidae	Dry mass	M = exp(a + b * log(L))	-4.591	2.543	11.0	47.0	Gowing and Recher (1984)	Not mentioned	Inferred, Diplopoda
Polydesmida	Opisotretidae	Dry mass	M = exp(a + b * log(L))	-4.591	2.543	11.0	47.0	Gowing and Recher (1984)	Not mentioned	Inferred, Diplopoda
Polydesmida	Pyrgodesmidae	Dry mass	M = exp(a + b * log(L))	-4.591	2.543	11.0	47.0	Gowing and Recher (1984)	Not mentioned	Inferred, Diplopoda
Polyxenida	Polyxenidae	Dry mass	M = exp(a + b * log(L))	-4.591	2.543	11.0	47.0	Gowing and Recher (1984)	Not mentioned	Inferred, Diplopoda
Pseudoscorpionida	All	Fresh mass	M = exp(a + b * log(L))	-1.892	2.515	0.86	2.10	Höfer and Ott (2009)	Edge of prosoma (without chelicerae) to edge of opisthosoma (excl spinnerets)	Group specific
Psocoptera	Uniden	Dry mass	M = a * (L)^b	0.014	3.115	1.50	3.15	Gruner (2003)	Tip of abdomen to end of head or carapace, excl. any appendages	Inferred, Psocoptera
Psocoptera	Psoquillidae	Dry mass	M = a * (L)^b	0.014	3.115	1.50	3.15	Gruner (2003)	Tip of abdomen to end of head or carapace, excl. any appendages	Inferred, Psocoptera
Psocoptera	Pachytroctidae	Dry mass	M = a * (L)^b	0.014	3.115	1.50	3.15	Gruner (2003)	Tip of abdomen to end of head or carapace, excl. any appendages	Inferred, Psocoptera
Psocoptera	Mesopsocidae	Dry mass	M = a * (L)^b	0.014	3.115	1.50	3.15	Gruner (2003)	Tip of abdomen to end of head or carapace, excl. any appendages	Inferred, Psocoptera
Psocoptera	Liposcelidae	Dry mass	M = a * (L)^b	0.014	3.115	1.50	3.15	Gruner (2003)	Tip of abdomen to end of head or carapace, excl. any appendages	Inferred, Psocoptera
Psocoptera	Ectopsocidae	Dry mass	M = a * (L)^b	0.014	3.115	1.50	3.15	Gruner (2003)	Tip of abdomen to end of head or carapace, excl. any appendages	Inferred, Psocoptera
Psocoptera	Epipsocidae	Dry mass	M = a * (L)^b	0.014	3.115	1.50	3.15	Gruner (2003)	Tip of abdomen to end of head or carapace, excl. any appendages	Inferred, Psocoptera

Psocoptera	Elipsocidae	Dry mass	M = a * (L)^b	0.014	3.115	1.50	3.15	Gruner (2003)	Tip of abdomen to end of head or carapace, excl. any appendages	Inferred, Psocoptera
Psocoptera	Hemipsocidae	Dry mass	M = a * (L)^b	0.014	3.115	1.50	3.15	Gruner (2003)	Tip of abdomen to end of head or carapace, excl. any appendages	Inferred, Psocoptera
Psocoptera	Lepidopsocidae	Dry mass	M = a * (L)^b	0.014	3.115	1.50	3.15	Gruner (2003)	Tip of abdomen to end of head or carapace, excl. any appendages	Inferred, Psocoptera
Psocoptera	Psyllipsocidae	Dry mass	M = a * (L)^b	0.014	3.115	1.50	3.15	Gruner (2003)	Tip of abdomen to end of head or carapace, excl. any appendages	Inferred, Psocoptera
Schizomida	Protoschizomidae	Fresh mass	M = exp(a + b * log(L))	-2.108	3.017	0.67	36	Höfer and Ott (2009)	Edge of prosoma (without chelicerae) to edge of opisthosoma (excl spinnerets)	Inferred, hunting spiders
Scolopendromorpha	Cryptopidae	Dry mass	M = exp(a + b * log(L))	-4.049	2.18	4	47	Gowing and Recher (1984)	Not mentioned	Inferred, Chilopoda
Symphyla	Scutegerillidae	Dry mass	M = a * (L)^b	0.035	2.173	0.9	17.6	Gruner (2003)	Tip of abdomen to end of head or carapace, excl. any appendages	Inferred, all insect taxa
Thysanoptera	Aeolothripidae	Dry mass	M = a * (L)^b	0.035	2.173	0.9	17.6	Gruner (2003)	Tip of abdomen to end of head or carapace, excl. any appendages	Inferred, all insect taxa
Thysanoptera	Phlaeothripidae	Dry mass	M = a * (L)^b	0.035	2.173	0.9	17.6	Gruner (2003)	Tip of abdomen to end of head or carapace, excl. any appendages	Inferred, all insect taxa
Thysanoptera	Thripidae	Dry mass	M = a * (L)^b	0.035	2.173	0.9	17.6	Gruner (2003)	Tip of abdomen to end of head or carapace, excl. any appendages	Inferred, all insect taxa
Thysanoptera	Merothripidae	Dry mass	M = a * (L)^b	0.035	2.173	0.9	17.6	Gruner (2003)	Tip of abdomen to end of head or carapace, excl. any appendages	Inferred, all insect taxa
Schizomida	Hubbardiidae	Fresh mass	M = exp(a + b * log(L))	-2.108	3.017	0.67	36	Höfer and Ott (2009)	Edge of prosoma (without chelicerae) to edge of opisthosoma (excl spinnerets)	Inferred, hunting spiders

Appendix Table 3: Dry-to-fresh mass conversion from the literature for transformation of dry body masses (DM) (from length-dry mass regression calculations) to fresh mass (FM).

Taxon	Equation FM[mg], DM[mg]	a	b	Reference	Regression specificity
All groups with dry-mass length-mass regressions (see Appendix Tab. 1)	FM = exp(a+b * log(DM))	0.6111	1.0213	Mercer et al. (2001)	Insecta

Appendix Table 4: Soil data (soil depth 0-10 cm) that were used in the PCA-analysis (baseline; 52 plots; no controls), resulting PCA scores of the soil data and a set of variables that describe the site conditions.

PlotID	Sand [proportion]	Silt [proportion]	Clay [proportion]	pH	Bulk Density [g/cm^3]	Total C [proportion]	PC axis 1	PC axis 2	PC axis 3	Slope [°]	Bare soil [proportion]	Gap fraction [proportion]	Altitude [m a.s.l.]
1	0.29	0.43	0.28	4.47	1.10	0.0184	0.01	-0.03	0.02	12.20	0.60	0.05	61.2
2	0.33	0.41	0.26	4.61	1.01	0.0229	0.03	0.10	0.10	12.60	0.25	0.04	46.9
3	0.35	0.37	0.27	4.47	1.03	0.0201	0.05	0.04	-0.02	7.80	0.10	0.07	40.5
4	0.27	0.41	0.32	4.40	0.93	0.0239	-0.06	0.11	-0.05	7.60	0.15	0.04	55.3
5	0.35	0.45	0.20	4.41	1.18	0.0182	0.06	-0.03	0.01	6.80	0.15	0.08	54.2
6	0.37	0.35	0.28	4.26	0.99	0.0167	0.06	0.02	-0.21	18.80	0.08	0.17	44.8
7	0.33	0.33	0.34	4.37	1.02	0.0200	0.02	0.00	-0.11	8.90	0.02	0.34	37.6
8	0.34	0.37	0.29	4.08	1.17	0.0123	0.04	-0.16	-0.28	5.10	0.20	0.04	62.6
9	0.39	0.31	0.30	5.02	1.13	0.0209	0.17	0.00	0.33	17.80	0.20	0.05	50.7
10	0.28	0.33	0.39	4.21	1.15	0.0194	-0.03	-0.14	-0.16	7.60	0.30	0.06	57.2
11	0.24	0.42	0.34	4.68	1.02	0.0168	0.00	-0.04	0.12	18.30	0.60	0.04	57.3
12	0.35	0.45	0.20	4.50	0.94	0.0244	0.02	0.21	0.04	16.80	0.01	0.59	53.0
13	0.11	0.54	0.35	4.38	1.24	0.0216	-0.17	-0.16	0.11	9.50	0.25	0.14	53.5
14	0.35	0.35	0.30	4.68	1.33	0.0104	0.16	-0.27	0.12	10.60	0.17	0.04	51.5
15	0.24	0.48	0.28	4.26	0.97	0.0370	-0.05	0.03	-0.14	8.20	0.01	NA	64.8
16	0.28	0.47	0.25	4.21	1.02	0.0191	-0.05	-0.07	0.16	8.70	0.05	0.17	54.1
17	0.25	0.44	0.31	4.49	1.26	0.0257	-0.04	-0.05	-0.12	5.10	0.07	0.12	54.6
18	0.25	0.43	0.31	4.30	1.04	0.0163	-0.31	0.15	0.22	6.60	0.08	0.10	51.9
19	0.08	0.55	0.37	4.41	1.05	0.0394	-0.10	0.18	0.11	2.40	0.10	0.30	48.2
20	0.24	0.47	0.29	4.56	0.91	0.0275	-0.08	-0.27	-0.04	2.00	0.10	0.12	42.3
21	0.19	0.43	0.39	4.28	1.30	0.0165	-0.13	-0.27	0.16	16.20	0.25	0.08	64.6
22	0.10	0.44	0.47	4.58	1.23	0.0178	-0.15	-0.15	0.15	20.80	0.10	0.10	44.2
23	0.14	0.53	0.33	4.40	1.29	0.0238	0.24	0.10	-0.01	20.90	0.10	0.03	53.5
24	0.53	0.23	0.24	4.61	1.04	0.0209	-0.10	-0.17	-0.28	6.80	0.08	0.05	53.6
25	0.20	0.43	0.37	4.07	1.06	0.0124	0.09	0.11	0.01	11.10	0.15	0.18	45.7
26	0.26	0.36	0.38	5.28	1.10	0.0228	0.35	-0.06	-0.06	6.40	NA	0.17	44.7
27	0.15	0.41	0.43	4.61	1.02	0.0258	-0.02	-0.14	0.24	1.80	NA	0.16	45.1
28	0.41	0.32	0.27	4.51	1.04	0.0245	0.11	0.01	0.22	21.30	0.10	0.06	67.1
29	0.60	0.18	0.22	4.59	1.20	0.0141	-0.25	0.07	-0.12	5.40	0.08	0.11	47.8
30	0.23	0.35	0.42	4.75	1.20	0.0227	0.06	0.07	-0.10	2.00	0.25	0.13	62.5
31	0.39	0.44	0.16	4.62	1.28	0.0235	0.17	0.09	-0.07	14.90	0.20	0.09	52.1
32	0.13	0.58	0.29	4.04	1.03	0.0265	-0.03	0.09	-0.01	1.00	0.05	0.11	52.5
33	0.38	0.34	0.28	4.37	1.01	0.0217	0.27	0.13	-0.21	4.60	0.20	0.06	60.5
34	0.46	0.33	0.21	4.53	0.98	0.0162	0.18	-0.04	-0.18	2.10	0.03	0.17	44.7

35	0.35	0.42	0.22	4.22	1.20	0.0312	-0.01	0.22	-0.09	0.60	0.08	0.10	44.4
36	0.60	0.26	0.14	4.31	1.06	0.0182	0.10	-0.02	0.06	2.80	0.40	0.05	47.2
37	0.50	0.22	0.27	4.27	1.19	0.0206	-0.30	-0.13	-0.28	0.20	0.20	0.04	45.1
38	0.35	0.42	0.23	4.33	0.89	0.0256	0.14	-0.09	-0.04	0.40	0.01	0.13	38.9
39	0.37	0.41	0.22	4.55	1.14	0.0172	0.12	-0.04	-0.02	2.70	0.05	0.17	43.3
40	0.05	0.42	0.53	3.97	0.97	0.0222	0.13	0.31	0.19	11.10	0.01	0.15	52.6
41	0.41	0.32	0.26	4.44	1.20	0.0169	0.03	-0.02	0.11	8.90	0.10	0.06	54.5
42	0.39	0.38	0.23	4.46	1.15	0.0165	-0.20	0.30	-0.01	7.50	0.05	0.18	46.6
43	0.46	0.38	0.17	4.78	0.91	0.0280	-0.20	-0.09	0.11	14.90	0.20	0.03	52.2
44	0.31	0.44	0.26	4.56	1.14	0.0198	-0.01	0.03	0.08	8.30	0.05	0.48	45.8
45	0.21	0.44	0.34	4.36	0.79	0.0351	0.01	-0.17	-0.01	2.80	0.00	0.16	42.8
46	0.10	0.47	0.44	4.46	1.11	0.0252	0.07	-0.22	0.16	17.50	0.20	0.06	47.2
47	0.28	0.39	0.33	4.59	1.02	0.0223	-0.29	0.15	-0.10	7.40	0.05	0.44	51.2
48	0.29	0.36	0.35	4.40	1.24	0.0184	-0.08	0.24	0.03	2.20	0.30	0.04	46.2
49	0.31	0.50	0.20	4.50	1.43	0.0152	-0.01	0.06	-0.11	9.10	0.30	0.16	43.0
50	0.13	0.51	0.37	4.09	0.93	0.0332	0.01	-0.03	0.02	3.50	0.00	0.43	47.9
51	0.28	0.53	0.20	4.43	0.88	0.0255	0.03	0.10	0.10	10.60	0.00	0.07	46.8
52	0.32	0.47	0.20	4.24	1.05	0.0196	0.05	0.04	-0.02	8.20	0.25	0.10	54.4

Appendix Table 5: Biotic data (baseline; 52 plots; no controls). S_{spp} = species richness, N = abundance, B = biomass, S_{fam} = family richness, LL inv. = leaf-litter invertebrates, HL inv. = herb-layer invertebrates.

PlotID	Plant S_{spp}	Plant N	Bird S_{spp}	Bird N	Bird B	LL inv. S_{fam}	LL inv. N	LL inv. B	HL inv. S_{fam}	HL inv. N	HL inv. B
1	13	220	0	0	0.00	5	7	3.61	4	12	27.32
2	24	482	4	6	233.43	7	11	5.32	8	33	168.57
3	15	739	3	12	131.70	6	8	12.45	22	69	137.97
4	20	482	4	15	308.93	30	210	141.08	15	35	373.86
5	16	996	4	14	150.02	3	11	0.96	8	18	65.77
6	16	586	5	9	291.51	11	24	23.77	7	17	479.79
7	15	607	2	10	90.48	4	14	7.40	25	42	235.60
8	16	675	4	8	372.08	7	10	22.61	5	15	102.00
9	11	274	8	21	1115.02	6	9	8.22	8	16	55.82
10	20	542	5	18	614.89	6	25	146.13	8	24	45.64
11	12	284	3	4	92.55	12	30	7.88	7	25	972.55
12	9	573	6	11	312.99	13	151	98.40	14	40	1133.81
13	17	203	4	10	335.13	6	18	14.00	3	5	327.33
14	16	361	4	16	588.90	5	9	2.57	6	12	116.43
16	14	554	4	14	289.06	13	39	37.00	22	50	116.08
17	20	718	6	14	810.48	12	23	48.73	16	48	473.72
18	19	603	7	19	1115.99	8	9	110.47	11	47	346.00
19	24	872	9	14	582.17	12	38	45.80	15	35	258.98
20	21	642	5	7	252.04	6	23	11.58	19	44	201.39
21	20	330	4	8	353.96	14	33	68.23	20	49	205.17
22	22	595	3	8	202.69	5	6	8.21	6	9	74.77
23	21	646	6	18	789.38	8	11	5.73	18	35	119.64
24	20	632	4	12	360.60	8	14	5.02	9	20	331.77
25	13	525	1	3	29.04	13	37	91.60	6	8	21.23
28	18	384	5	15	570.60	12	19	45.60	19	29	36.28
29	20	444	6	13	565.84	10	20	33.78	5	10	88.11
30	20	750	4	12	296.83	4	4	12.84	15	38	165.46
31	19	438	7	10	317.63	13	27	31.21	4	9	118.56
32	23	507	2	4	56.84	12	46	21.70	10	31	147.92
33	13	734	5	8	467.71	4	9	2.15	6	25	102.52
34	14	474	7	25	701.73	6	29	34.44	12	39	805.17
35	21	492	3	4	118.86	3	35	35.09	14	53	233.24
36	15	594	4	5	223.50	6	14	10.69	4	13	101.92
37	13	1092	11	21	1244.57	6	7	0.76	6	13	193.15
38	20	851	7	18	542.43	15	42	53.03	12	18	268.28
39	19	414	4	14	224.08	6	9	25.17	11	17	57.79
40	21	304	5	13	342.32	8	20	17.41	9	22	512.67
41	8	120	2	4	74.96	14	83	94.65	15	27	39.08
42	10	399	2	3	27.46	9	23	11.30	23	68	685.27
43	18	340	2	6	50.18	3	4	1.18	7	13	106.69
44	17	514	6	15	330.03	11	112	78.22	9	19	289.24
45	16	379	7	32	676.98	16	83	149.17	27	54	746.31
46	17	268	3	6	128.98	26	224	180.06	5	36	159.10
47	16	254	1	1	27.80	14	25	9.33	6	16	610.80
48	14	230	3	6	140.92	3	5	2.47	5	6	30.08
49	12	223	3	6	261.76	3	7	5.05	13	23	145.71
50	14	369	3	7	221.96	17	69	49.84	22	46	302.90
51	26	682	5	15	420.08	16	63	90.36	19	39	78.98
52	19	455	4	8	156.66	1	3	0.61	6	13	63.74

Appendix Table 6: Summary tables from linear and generalized linear models testing the effects of plot size and tree diversity on the environmental variables. In case of overdispersion, we used the negative binomial distribution for modelling. Asterisks denote significance levels: * $p < 0.05$; ** $p < 0.01$; *** $p < 0.001$. TD = tree diversity, PS = plot size, S_{spp} = species richness, N = abundance, S_{fam} = family richness, B = biomass, LL inv. = leaf-litter invertebrates, HL inv. = herb-layer invertebrates.

Response	Fixed effects	Estimate	Std. Error	t-value	p-value		R^2
PCaxis1	TD	-0.046	0.034	-1.383	0.170		0.046
	PS	-0.002	0.014	0.127	0.899		
	TD: PS	0.004	0.006	0.726	0.470		
PCaxis 2	TD	0.063	0.033	1.925	0.057		0.080
	PS	0.047	0.014	3.290	0.001	**	
	TD: PS	-0.013	0.006	-2.138	0.035	*	
PCaxis 3	TD	0.020	0.033	0.593	0.555		0.056
	PS	0.022	0.014	1.545	0.126		
	TD: PS	-0.001	0.006	-0.089	0.929		
Slope	TD	-3.356	1.387	-2.413	0.018	*	0.064
	PS	-1.688	0.596	-2.833	0.006	**	
	TD: PS	0.699	0.251	2.779	0.007	**	
Bare soil	TD	-0.473	0.310	-1.526	0.130		0.008
	PS	-0.047	0.133	-0.352	0.726		
	TD: PS	0.073	0.056	1.299	0.197		
Gap fraction	TD	0.495	0.174	2.851	0.005	**	0.084
	PS	0.062	0.075	0.834	0.406		
	TD: PS	-0.079	0.031	-2.506	0.014	*	
Altitude	TD	-0.309	1.625	-0.190	0.849		-0.025
	PS	-0.468	0.698	-0.671	0.504		
	TD: PS	0.067	0.294	0.229	0.820		
Plant S_{spp} (nb)	TD	-0.157	0.065	-2.419	0.016	*	0.078
	PS	-0.066	0.028	-2.388	0.017	*	
	TD: PS	0.032	0.012	2.764	0.006	**	
Plant N	TD	0.048	0.100	0.476	0.634		0.021
	PS	0.010	0.043	0.225	0.822		
	TD: PS	-0.001	0.018	-0.075	0.940		
Bird S_{spp}	TD	-0.182	0.113	-1.611	0.107		0.056
	PS	-0.104	0.049	-2.106	0.035	*	
	TD: PS	0.040	0.020	1.989	0.047	*	
Bird N	TD	-0.130	0.336	7.891	0.368		0.009
	PS	-0.048	0.061	-0.783	0.433		
	TD: PS	0.024	0.026	0.946	0.344		
Bird B	TD	-0.226	0.225	-1.006	0.317		0.026
	PS	-0.176	0.097	-1.826	0.071		
	TD: PS	0.061	0.041	1.492	0.139		
LL inv. S_{fam}	TD	0.037	0.133	0.283	0.777		0.048
	PS	-0.072	0.059	-1.226	0.220		
	TD: PS	0.002	0.024	0.078	0.938		
LL inv. N	TD	0.049	0.234	1.829	0.067		0.037
	PS	0.195	0.101	1.937	0.053		
	TD: PS	-0.087	0.043	-2.048	0.041	*	
LL inv. B	TD	0.377	0.343	1.097	0.275		0.089
	PS	-0.150	0.148	-1.020	0.311		
	TD: PS	-0.060	0.062	-0.967	0.336		
HL inv. S_{fam}	TD	0.202	0.123	1.644	0.100		0.071

	PS	0.055	0.055	1.001	0.317	
	TD: PS	-0.023	0.022	-1.010	0.312	
HL inv. N	TD	0.243	0.136	1.791	0.073	0.053
	PS	0.089	0.059	1.512	0.131	
	TD: PS	-0.034	0.025	-1.375	0.169	
HL inv. B	TD	0.008	0.237	0.035	0.972	0.001
	PS	-0.011	0.102	-0.112	0.911	
	TD: PS	-0.002	0.043	-0.039	0.969	

Appendix Table 7: List of plant species (baseline; 52 plots; no controls). N = abundance, F = frequence (number of plots present on).

Species	Family	N	F
Adiantum latifolium Lam.	Pteridaceae	264	32
Ageratum conyzoides (L.) L.*	Compositae	381	16
Alternanthera sessilis (L.) R.Br. ex DC.	Amaranthaceae	57	4
Asplenium normale D. Don	Aspleniaceae	109	17
cf. *Asplenium* spec.	Aspleniaceae	3	2
Asystasia gangetica (L.) T.Anderson*	Acanthaceae	5253	49
Bauhinia semibifida Roxb.	Fabaceae	1	1
Blechnum orientale L.	Blechnaceae	8	2
Breynia cernua (Poir.) Müll.Arg.	Phyllanthaceae	7	4
Centotheca lappacea (L.) Desv.	Poaceae	1714	42
Centrosema pubescens Benth.*	Fabaceae	38	5
Chromolaena odorata (L.) R.M.King & H.Rob.*	Compositae	9	5
Cleome rutidosperma DC.*	Cleomaceae	5	1
Clerodendrum spec.	Lamiaceae	2	2
Clidemia hirta (L.) D. Don*	Melastomataceae	3233	50
Commelina diffusa Burm.f.*	Commelinaceae	7	1
Coptosapelta flavescens Korth.	Rubiaceae	2	1
Crassocephalum crepidioides (Benth.) S.Moore*	Compositae	3	2
Croton argyratus Blume	Euphorbiaceae	1	1
Croton hirtus L'Hér.*	Euphorbiaceae	24	5
Cuphea carthagenensis (Jacq.) J.F.Macbr.*	Lythraceae	8	2
Cyclosorus heterocarpus (Blume) Ching	Thelypteridaceae	54	7
Cyclosorus megaphyllus Ching	Thelypteridaceae	1625	41
Cyperus diffusus Vahl	Cyperaceae	165	28
Cyrtococcum patens (L.) A.Camus	Poaceae	1286	40
Dicranopteris linearis (Burm. f.) Underw.	Gleicheniaceae	9	1
Dioscorea alata L.	Dioscoreaceae	3	2
Elaeis guineensis Jacq.*	Arecaceae	131	30
fern spec. 01		1	1
cf. *Ficus* spec.	Moraceae	1	1
Ficus cf. *variegata* Blume	Moraceae	19	7
grass spec. 01	Cyperaceae	4	1
grass spec. 02	Poaceae	18	6
herb spec. 01		1	1
herb spec. 02		2	1
herb spec. 03		2	1
herb spec. 04		1	1
herb spec. 05		1	1
Hyptis capitata Jacq.*	Lamiaceae	6	1
Imperata cylindrica (L.) Raeusch*	Poaceae	85	10
Lantana camara L.*	Verbenaceae	2	1
Lindernia crustacea (L.) F.Muell.	Linderniaceae	8	4
Lindernia diffusa (L.) Wettst.*	Linderniaceae	1	1
Ludwigia octovalvis (Jacq.) P.H.Raven*	Onagraceae	4	2
Lygodium circinatum (Burm. f.) Sw.	Lygodiaceae	91	23
Mallotus peltatus (Geiseler) Müll.Arg.	Euphorbiaceae	5	1
Melastoma malabathricum L.*	Melastomataceae	66	23
Merremia umbellata (L.) Hallier f.	Convolvulaceae	78	7
Microlepia speluncae (L.) T. Moore	Dennstaedtiaceae	76	22
Mikania micrantha (L.) Willd.*	Compositae	81	17
Mimosa cf. *pudica* L.*	Fabaceae	13	4
Nephrolepis cf. *acutifolia* (Desv.) Christ	Nephrolepidaceae	2299	42
Oplismenus compositus (L.) P.Beauv.	Poaceae	38	2
Ottochloa nodosa (Kunth) Dandy	Poaceae	1962	44
Oxalis barrelieri L.*	Oxalidaceae	6	1
Panicum cf. *laxum* Sw.*	Poaceae	2194	29
Paspalum cf. *conjugatum* P.J.Bergius*	Poaceae	1584	49
Peperomia pellucida (L.) Kunth*	Piperaceae	6	1

Pericampylus glaucus (Lam.) Merr.	Menispermaceae	1	1
Phyllanthus cf. *niruri* L.	Phyllanthaceae	19	4
Pronephrium triphyllum (Sw.) Holttum	Thelypteridaceae	35	4
Pteris cf. *armata* C. Presl	Pteridaceae	50	4
Pteris ensiformis Burm. f.	Pteridaceae	1	1
Pueraria phaseoloides (Roxb.) Benth.	Fabaceae	5	2
Salvia occidentalis Sw.	Lamiaceae	3	1
Schizostachyum sp.	Poaceae	1	1
Scleria levis Retz.	Cyperaceae	283	37
seedling spec. 1		1	1
Selaginella willdenowii (Desv. ex Poir.) Baker	Selaginellaceae	126	14
Solanum jamaicense Mill.*	Solanaceae	12	6
Spermacoce alata Aubl.*	Rubiaceae	1814	32
Stenochlaena palustris (Burm. f.) Bedd.	Blechnaceae	14	2
Strombosia javanica Thwaites	Olacaceae	11	1
Taenitis blechnoides (Willd.) Sw.	Pteridaceae	452	23
Tectaria vasta (Blume) Copel.	Tectariaceae	21	6
Urceola brachysepala Hook.f.	Apocynaceae	29	3
Urceola spec.	Apocynaceae	108	8
woody spec. 01		1	1
woody spec. 02		3	2
woody spec. 03		1	1
woody spec. 04		34	3
woody spec. 05		12	1
woody spec. 06		1	1
woody spec. 07		1	1
woody spec. 08		1	1
woody spec. 09		1	1
woody spec. 10		2	1
woody spec. 11		1	1
woody spec. 12		1	1
woody spec. 13		2	1
woody spec. 14		1	1
woody spec. 15		1	1

Species: * = alien species

Appendix Table 8: List of bird species recorded within a 75 m radius around the centre of each plot with information on diet and main habitat. Total abundance, total biomass and frequence of the bird species from the baseline survey (52 plots, no controls) as well as from year 1 (56 plots, control plots included) are given. Diet: Invertebrate = invertebrates; PlantSeed = plants and seeds; FruiNect = fruits and nectar (≥ 50% of their diet consists of fruit and/or nectar but they also feed on invertebrates, plants or seeds); VertFishScav = vertebrates, fish, carrion; Omnivore = omnivore (score of ≤ 50 in all four categories) (for detailed information on categories see Wilman et al. (2014)). Habitat: main natural habitat: 1 = primary and old secondary forests interior; 2 = forest gaps, edges, or upper canopy; 3 = little wooded and cultivated areas. All species are listed as 'Least Concern' (IUCN, 2015). Diet and biomass data was taken from Wilman et al. (2014). N = abundance, B = biomass, F = frequence (number of plots present on).

					**Baseline**			**Year 1**		
Species	Family	Diet	Habitat	Habitat source	N	B [g/75 m radius]	F	N	B [g/75 m radius]	F
Amaurornis phoenicurus	Rallidae	Omnivore	3	Beukema et al. (2007)	10	1440.00	5	-	-	-
Celeus brachyurus	Picidae	Invertebrate	2	Thiollay (1995)	1	85.94	1	-	-	-
Centropus sinensis	Cuculidae	Omnivore	3	Thiollay (1995)	1	280.70	1	1	280.70	1
Chalcophaps indica	Columbidae	Omnivore	1	Thiollay (1995)	9	1228.86	7	10	955.78	7
Coturnix chinesis	Phasianidae	PlantSeed	3	Pappas (2001)	-	-	-	1	40.41	1
Dicaeum trigonostigma	Dicaeidae	FruiNect	2	Thiollay (1995)	16	113.60	13	20	127.80	16
Eurystomus orientalis	Coraciidae	Invertebrate	2	Thiollay (1995)	2	286.04	2	-	-	-
Geopelia striata	Columbidae	PlantSeed	3	Thiollay (1995)	8	452.80	6	41	1811.20	18
Halcyon smyrnensis	Alcedinidae	VertFishScav	3	Thiollay (1995)	33	2924.80	22	19	1736.60	13
Lanius schach	Laniidae	Invertebrate	3	Yosef et al. (2015)	-	-	-	2	103.02	2
Lonchura punctulata	Estrildidae	PlantSeed	3	Thiollay (1995)	-	-	-	7	40.80	3
Megalaima haemacephala	Ramphastidae	FruiNect	3	Thiollay (1995)	2	88.98	2	-	-	-
Merops viridis	Meropidae	Invertebrate	3	Thiollay (1995)	3	34.80	1	-	-	-
Orthotomus atrogularis	Sylviidae	Invertebrate	3	Thiollay (1995)	-	-	-	1	7.68	1
Orthotomus ruficeps	Sylviidae	Invertebrate	3	Thiollay (1995)	61	429.30	27	45	210.60	20
Orthotomus sericeus	Sylviidae	Invertebrate	2	Thiollay (1995)	21	216.00	14	1	10.80	1
Prinia familiaris	Cisticolidae	Invertebrate	3	Thiollay (1995)	241	1810.16	48	252	1539.12	50
Pycnonotus aurigaster	Pycnonotidae	Omnivore	3	Thiollay (1995)	31	1019.59	14	6	221.65	3
Pycnonotus goiavier	Pycnonotidae	FruiNect	3	Thiollay (1995)	191	4698.20	38	198	4281.20	45

Pycnonotus plumosus	Pycnonotidae	FruiNect	3	Thiollay (1995)	14	453.05	7	6	174.25	4
Rhipidura javanica	Rhipiduridae	Invertebrate	3	Robson (2015)	2	25.00	2	5	62.50	4
Spilornis cheela	Accipitridae	VertFishScav	2	Thiollay (1995)	1	597.74	1	8	4781.92	6
Spizaetus cirrhatus	Accipitridae	VertFishScav	3	Thiollay (1995)	-	-	-	1	1475.12	1
Stigmatopelia chinensis	Columbidae	PlantSeed	3	Thiollay (1995)	8	1272.00	6	64	8904.00	28
Todiramphus chloris	Alcedinidae	Invertebrate	3	Thiollay (1995)	14	859.17	10	61	3634.95	32
Treron vernans	Columbidae	FruiNect	2	Thiollay (1995)	3	396.00	3	-	-	-

Appendix Table 9: List of families of leaf-litter invertebrates recorded on the plots. Total abundance, total biomass and frequence of the invertebrate families from the baseline survey (52 plots, no controls) as well as from year 1 (56 plots, control plots included) are given. Each family was assigned a feeding type which was taken from the literature. Data on biomass was calculated based on Appendix Tab. 2. N = abundance, B = biomass, F = frequence (number of plots present on).

				**Baseline**			**Year 1**		
Family	Order	Feeding type (FT)	FT Source	N	B [mg/m^2]	F	N	B [mg/m^2]	F
Blattellidae	Blattodea	Detritivore	CSIRO (1991)	7	18.11	5	12	15.18	10
Corinnidae	Araneae	Predator	Jocqué and Dippenaar-Schoeman (2006)	17	79.22	14	15	64.10	14
Formicidae	Hymenoptera	Omnivore	CSIRO (2013)	1291	562.23	50	1551	1163.63	53
Gryllidae	Orthoptera	Omnivore	Schowalter (2009)	6	25.86	6	16	27.30	12
Oxyopidae	Araneae	Predator	Jocqué and Dippenaar-Schoeman (2006)	7	36.46	7	11	23.57	10
Theridiidae	Araneae	Predator	Jocqué and Dippenaar-Schoeman (2006)	45	43.31	23	39	31.61	23
Theridiosomatidae	Araneae	Predator	Jocqué and Dippenaar-Schoeman (2006)	19	4.36	13	1	0.17	1
Linyphiidae	Araneae	Predator	Jocqué and Dippenaar-Schoeman (2006)	28	9.91	18	64	20.29	23
Lycosidae	Araneae	Predator	Jocqué and Dippenaar-Schoeman (2006)	44	126.11	23	58	225.67	28
Mecistocephalidae	Geophilomorpha	Predator	Colloff et al. (2005)	-	-	-	3	39.98	3
Oonopidae	Araneae	Predator	Jocqué and Dippenaar-Schoeman (2006)	34	12.79	15	13	4.11	8
Phalacridae	Coleoptera	Fungivore	CSIRO (1991)	36	8.67	3	18	1.38	12
Staphylinidae	Coleoptera	Predator	CSIRO (1991)	113	24.53	20	34	7.27	17

Cicadellidae	Hemiptera	Herbivore	CSIRO (1991)	1	1.24	1	4	1.41	3
Gnaphosidae	Araneae	Predator	Jocqué and Dippenaar-Schoeman (2006)	36	16.40	24	4	3.25	4
Japygidae	Diplura	Predator	CSIRO (1991)	9	13.70	5	10	10.84	7
Mysmenidae	Araneae	Predator	Jocqué and Dippenaar-Schoeman (2006)	30	2.91	16	-	-	-
Acrididae	Orthoptera	Detritivore	CSIRO (1991)	-	-	-	2	233.96	1
Scolytinae	Coleoptera	Detritivore	CSIRO (1991)	3	0.74	3	1	0.11	1
Tetrigidae	Orthoptera	Detritivore	Kocarek et al. (2011)	4	14.90	4	3	37.38	2
Dipsocoridae	Hemiptera	Predator	CSIRO (2013)	22	2.66	10	14	1.40	9
Geophilidae	Geophilomorpha	Predator	CSIRO (1991)	16	207.48	10	1	12.15	1
Pyrgodesmidae	Polydesmida	Detritivore	CSIRO (1991)	70	62.15	20	65	63.11	20
Corylophidae	Coleoptera	Detritivore	CSIRO (1991)	-	-	-	6	1.23	5
Elateridae	Coleoptera	Herbivore	CSIRO (1991)	2	0.08	2	3	0.25	2
Blaberidae	Blattodea	Detritivore	CSIRO (1991)	10	19.14	7	9	3.59	6
Carabidae	Coleoptera	Herbivore	CSIRO (1991)	7	9.22	5	19	6.86	12
Dalodesmidae	Polydesmida	Detritivore	CSIRO (1991)	3	6.40	1	-	-	-
Aderidae	Coleoptera	Herbivore	CSIRO (1991)	-	-	-	10	2.10	5
Cydnidae	Hemiptera	Herbivore	CSIRO (1991)	8	3.42	2	11	3.33	6
Gracillariidae	Lepidoptera	Herbivore	CSIRO (1991)	-	-	-	3	0.97	3
Henicopidae	Lithobiomorpha	Predator	Colloff et al. (2005)	16	18.57	6	-	-	-
Labiidae	Dermaptera	Omnivore	CSIRO (1991)	7	26.39	7	-	-	-
Largidae	Hemiptera	Herbivore	CSIRO (1991)	6	2.39	4	-	-	-
Mantidae	Mantodea	Predator	CSIRO (1991)	3	0.71	3	-	-	-
Oniscidae	Isopoda	Detritivore	Zimmer (2002)	1	0.18	1	-	-	-
Nabidae	Hemiptera	Predator	CSIRO (1991)	-	-	-	15	27.77	10
Pentatomidae	Hemiptera	Herbivore	CSIRO (1991)	-	-	-	5	543.95	4
Philosciidae	Isopoda	Detritivore	Zimmer (2002)	44	115.33	17	10	20.44	9
Salticidae	Araneae	Predator	Jocqué and Dippenaar-Schoeman (2006)	24	59.81	18	22	85.07	16
Scelionidae	Hymenoptera	Omnivore	CSIRO (1991)	-	-	-	2	0.10	2
Scutigerellidae	Symphyla	Detritivore	CSIRO (1991)	4	1.49	4	11	4.73	8
Phlaeothripidae	Thysanoptera	Fungivore	CSIRO (2013)	7	1.39	6	9	2.45	6
Protoschizomidae	Schizomida	Predator	Harvey (2015)	1	0.84	1	-	-	-

Silvanidae	Coleoptera	Predator	CSIRO (1991)	-	-	-	10	1.64	8
Pseudococcidae	Hemiptera	Herbivore	Ben-Dov (1994)	1	0.39	1	-	-	-
Sironidae	Opiliones	Predator	Pinto-da-Rocha et al. (2007)	1	14.35	1	-	-	-
Tenebrionidae	Coleoptera	Detritivore	CSIRO (1991)	13	56.57	9	-	-	-
Termitidae	Isoptera	Detritivore	CSIRO (1991)	10	8.93	1	1	1.48	1
Tetrablemmidae	Araneae	Predator	Jocqué and Dippenaar-Schoeman (2006)	10	1.50	6	2	0.51	2
Polyxenidae	Polyxenida	Detritivore	CSIRO (1991)	5	0.59	5	12	4.52	7
Rhinotermitidae	Isoptera	Detritivore	CSIRO (1991)	3	1.11	2	-	-	-
Thomisidae	Araneae	Predator	Jocqué and Dippenaar-Schoeman (2006)	13	39.57	10	12	22.65	8
Colydiidae	Coleoptera	Fungivore	CSIRO (1991)	2	0.33	2	-	-	-
Discolomidae	Coleoptera	Fungivore	CSIRO (1991)	-	-	-	1	0.39	1
Hydraenidae	Coleoptera	Herbivore	CSIRO (1991)	2	0.26	1	-	-	-
Miridae	Hemiptera	Herbivore	CSIRO (1991)	2	0.35	2	-	-	-
Pselaphidae	Coleoptera	Predator	CSIRO (1991)	-	-	-	7	1.48	7
Pachytroctidae	Psocoptera	Detritivore	Gruner (2004)	1	0.07	1	3	0.14	2
Schizopteridae	Hemiptera	Predator	Reagan and Waide (1996)	6	0.18	5	7	0.16	5
Reduviidae	Hemiptera	Predator	CSIRO (1991)	8	227.19	6	2	63.06	2
Zodariidae	Araneae	Predator	Jocqué and Dippenaar-Schoeman (2006)	-	-	-	5	19.51	5
Dignathodontidae	Geophilomorpha	Predator	CSIRO (1991)	1	0.25	1	1	0.25	1
Neobisiidae	Pseudoscorpion	Predator	Eisenbeis (2006)	1	0.29	1	1	0.24	1
Opisotretidae	Polydesmida	Detritivore	CSIRO (1991)	6	4.81	4	-	-	-
Cryptodesmidae	Polydesmida	Detritivore	David (2009)	1	0.70	1	2	2.10	2
Nitidulidae	Coleoptera	Herbivore	CSIRO (1991)	2	1.69	2	4	0.55	2
Aeolothripidae	Thysanoptera	Omnivore	CSIRO (2013)	-	-	-	2	0.27	2
Scydmaenidae	Coleoptera	Predator	CSIRO (1991)	3	0.08	1	4	0.69	3
Blattidae	Blattodea	Detritivore	CSIRO (1991)	8	7.08	7	2	0.66	2
Delphacidae	Hemiptera	Herbivore	CSIRO (1991)	2	0.47	2	-	-	-
Lagrioidinae	Coleoptera	Herbivore	Costa et al. (1995)	-	-	-	1	0.26	1
Lygaeidae	Hemiptera	Herbivore	CSIRO (1991)	6	1.32	4	-	-	-
Drosophilidae	Diptera	Detritivore	McAlpine (1981)	1	0.50	1	-	-	-
Araneidae	Araneae	Predator	Jocqué and Dippenaar-Schoeman (2006)	3	7.66	3	7	25.20	7

Dryinidae	Hymenoptera	Parasite	Goulet and Huber (1993)	1	0.40	1	1	0.30	1
Glomeridae	Glomerida	Detritivore	David (2009)	4	11.06	3	-	-	-
Sparassidae	Araneae	Predator	Jocqué and Dippenaar-Schoeman (2006)	-	-	-	3	1.56	3
Ctenidae	Araneae	Predator	Jocqué and Dippenaar-Schoeman (2006)	2	15.84	2	4	16.92	3
Haplodesmidae	Polydesmida	Detritivore	CSIRO (1991)	-	-	-	6	1.65	4
Chrysomelidae	Coleoptera	Detritivore	CSIRO (1991)	2	13.02	2	15	1.98	2
Psoquillidae	Psocoptera	Detritivore	Gruner (2004)	3	0.96	3	-	-	-
Olpiidae	Pseudoscorpion	Predator	CSIRO (1991)	1	0.35	1	-	-	-
Ectopsocidae	Psocoptera	Detritivore	Gruner (2004)	-	-	-	12	0.49	3
Tetragnathidae	Araneae	Predator	Jocqué and Dippenaar-Schoeman (2006)	-	-	-	8	21.82	6
Labiduridae	Dermaptera	Omnivore	CSIRO (1991)	1	4.35	1	-	-	-
Pholcidae	Araneae	Predator	Jocqué and Dippenaar-Schoeman (2006)	1	0.21	1	-	-	-
Spongiphoridae	Dermaptera	Omnivore	CSIRO (1991)	22	84.49	2	-	-	-
Aphididae	Hemiptera	Herbivore	CSIRO (1991)	1	0.06	1	4	0.60	3
Heteroceridae	Coleoptera	Herbivore	Clarke (1973)	-	-	-	1	4.89	1
Liposcelidae	Psocoptera	Detritivore	Gruner (2004)	1	0.24	1	3	0.05	1
Tridactylidae	Orthoptera	Herbivore	CSIRO (1991)	1	2.54	1	-	-	-
Ptiliidae	Coleoptera	Fungivore	CSIRO (1991)	1	0.03	1	1	0.03	1
Tingidae	Hemiptera	Herbivore	CSIRO (1991)	3	0.30	2	-	-	-
Ceratocombidae	Hemiptera	Predator	CSIRO (2013)	-	-	-	1	0.01	1
Stratiomydae	Diptera	Detritivore	CSIRO (1991)	1	4.54	1	-	-	-
Armadillidae	Isopoda	Detritivore	Zimmer (2002)	3	29.15	1	-	-	-
Tettigoniidae	Orthoptera	Omnivore	CSIRO (1991)	1	3.11	1	-	-	-
Myrmeleontidae	Neuroptera	Predator	Gepp and Hölzel (1989)	1	2.00	1	-	-	-
Curculionidae	Coleoptera	Detritivore	CSIRO (1991)	-	-	-	1	1.62	1
Scarabaeidae	Coleoptera	Detritivore	CSIRO (1991)	-	-	-	1	8.66	1
Cryptopidae	Scolopendromorpha	Predator	Colloff et al. (2005)	4	13.23	2	-	-	-
Campodeidae	Diplura	Omnivore	CSIRO (1991)	1	0.34	1	-	-	-
Epipsocidae	Psocoptera	Detritivore	Gruner (2004)	2	0.08	1	-	-	-
Paradoxosomatidae	Polydesmida	Detritivore	David (2009)	1	0.54	1	-	-	-
Platyrhacidae	Polydesmida	Detritivore	David (2009)	1	22.35	1	-	-	-

Hubbardiidae	Schizomida	Predator	(Harvey, 2015)	1	9.90	1	-	-	-
Phalangodidae	Opiliones	Predator	Pinto-da-Rocha et al. (2007)	4	7.15	2	-	-	-
Mesopsocidae	Psocoptera	Detritivore	Gruner (2004)	1	0.05	1	-	-	-
Hemipsocidae	Psocoptera	Detritivore	Gruner (2004)	-	-	-	3	0.09	1
Pyralidae	Lepidoptera	Herbivore	Stehr (2005)	-	-	-	1	1.82	1
Gelechiidae	Lepidoptera	Herbivore	Stehr (2005)	-	-	-	1	0.58	1
Anisolabididae	Dermaptera	Omnivore	Capinera (2008)	-	-	-	3	76.89	1

Appendix Table 10: List of families of herb-layer invertebrates recorded on the plots. Total abundance, total biomass and frequence of the invertebrate families from the baseline survey (52 plots, no controls) as well as from year 1 (56 plots, control plots included) are given. Each family was assigned a feeding type which was derived from the literature. Data on biomass was calculated based on Appendix Tab. 2. N = abundance, B = biomass, F = frequence (number of plots present on).

				Baseline			Year 1		
Order	Family	Feeding type (FT)	FT Source	N	B [mg/m^2]	F	N	B [mg/m^2]	F
Hemiptera	Aphididae	Herbivore	CSIRO (1991)	14	0.81	5	38	3.32	21
Araneae	Corinnidae	Predator	Jocqué and Dippenaar-Schoeman (2006)	-	-	-	7	7.45	7
Hymenoptera	Formicidae	Omnivore	CSIRO (2013)	91	68.48	34	255	213.86	52
Orthoptera	Gryllidae	Omnivore	Schowalter (2009)	499	3064.30	51	764	7354.87	54
Araneae	Salticidae	Predator	Jocqué and Dippenaar-Schoeman (2006)	23	92.96	15	36	140.86	25
Diptera	Culicidae	Predator	CSIRO (1991)	26	21.68	20	5	1.21	5
Diptera	Tipulidae	Detritivore	CSIRO (1991)	23	25.59	14	5	2.75	5
Hymenoptera	Braconidae	Parasite	Goulet and Huber (1993)	8	2.94	8	32	15.95	17
Hymenoptera	Scelionidae	Omnivore	CSIRO (1991)	25	3.62	12	89	10.69	37
Orthoptera	Acrididae	Detritivore	CSIRO (1991)	58	6282.27	24	100	10985.43	38
Hemiptera	Cicadellidae	Herbivore	CSIRO (1991)	96	123.60	26	146	196.70	40
Hymenoptera	Eucoilidae	Omnivore	CSIRO (1991)	-	-	-	2	0.08	2
Araneae	Linyphiidae	Predator	Jocqué and Dippenaar-Schoeman (2006)	2	3.95	2	17	30.45	16
Mantodea	Mantidae	Predator	CSIRO (1991)	8	298.35	7	14	527.72	12
Hemiptera	Miridae	Herbivore	CSIRO (1991)	30	5.29	18	50	11.17	16
Araneae	Oxyopidae	Predator	Jocqué and Dippenaar-Schoeman (2006)	79	829.09	30	91	574.37	38
Araneae	Sparassidae	Predator	Jocqué and Dippenaar-Schoeman (2006)	-	-	-	4	22.34	4
Araneae	Lycosidae	Predator	Jocqué and Dippenaar-Schoeman (2006)	46	461.74	26	85	1273.62	36
Blattodea	Blattellidae	Detritivore	CSIRO (1991)	15	178.96	15	7	8.19	5
Hymenoptera	Diapriidae	Omnivore	CSIRO (1991)	3	0.46	3	-	-	-
Orthoptera	Tettigoniidae	Omnivore	CSIRO (1991)	19	703.32	11	20	2320.63	16
Thysanoptera	Aeolothripidae	Omnivore	CSIRO (2013)	-	-	-	7	0.25	7

Hemiptera	Aleyrodidae	Herbivore	Pappas et al. (2013)	-	-	-	23	1.80	14
Diptera	Cecidomyiidae	Detritivore	CSIRO (1991)	9	0.27	6	72	3.53	34
Hemiptera	Dictyopharidae	Herbivore	Wilson et al. (1994)	-	-	-	2	20.33	2
Hymenoptera	Mymaridae	Parasite	Goulet and Huber (1993)	2	0.06	2	59	1.26	27
Thysanoptera	Thripidae	Herbivore	CSIRO (1991)	-	-	-	22	1.28	13
Araneae	Theridiidae	Predator	Jocqué and Dippenaar-Schoeman (2006)	28	91.57	21	8	3.24	7
Araneae	Thomisidae	Predator	Jocqué and Dippenaar-Schoeman (2006)	22	76.50	14	8	22.93	6
Coleoptera	Staphylinidae	Predator	CSIRO (1991)	4	3.11	4	3	1.67	3
Diptera	Asteiidae	Fungivore	CSIRO (1991)	22	3.30	9	5	0.55	3
Diptera	Ceratopogonidae	Detritivore	McAlpine (1981)	11	2.01	8	11	0.62	6
Diptera	Phoridae	Detritivore	CSIRO (1991)	2	0.10	2	-	-	-
Diptera	Piophilidae	Detritivore	McAlpine (1981)	3	0.21	3	-	-	-
Diptera	Psychodidae	Detritivore	CSIRO (1991)	4	0.13	4	1	0.03	1
Hemiptera	Anthocoridae	Herbivore	CSIRO (1991)	10	1.57	2	-	-	-
Hemiptera	Delphacidae	Herbivore	CSIRO (1991)	53	71.70	21	23	12.66	18
Hymenoptera	Trichogrammatidae	Omnivore	CSIRO (2013)	1	0.00	1	15	0.18	13
Lepidoptera	Thyrididae	Herbivore	CSIRO (1991)	4	21.64	4	-	-	-
Lithobiomorpha	Henicopidae	Predator	Colloff et al. (2005)	1	0.21	1	-	-	-
Araneae	Araneidae	Predator	Jocqué and Dippenaar-Schoeman (2006)	12	70.62	9	2	4.00	2
Hemiptera	Cixiidae	Herbivore	CSIRO (1991)	-	-	-	14	3.91	10
Hymenoptera	Encyrtidae	Parasite	Goulet and Huber (1993)	1	0.04	1	18	0.53	16
Hymenoptera	Eulophidae	Parasite	Goulet and Huber (1993)	12	1.86	9	22	2.75	17
Hemiptera	Nabidae	Predator	CSIRO (1991)	2	1.06	2	27	88.67	13
Thysanoptera	Phlaeothripidae	Fungivore	CSIRO (2013)	6	0.55	3	11	1.39	6
Lepidoptera	Pyralidae	Herbivore	Stehr (2005)	1	8.15	1	1	1.50	1
Orthoptera	Tetrigidae	Detritivore	Kocarek et al. (2011)	36	1010.79	22	37	978.99	23
Araneae	Theridiosomatidae	Predator	Jocqué and Dippenaar-Schoeman (2006)	8	1.14	7	75	24.19	34
Coleoptera	Aderidae	Herbivore	CSIRO (1991)	4	2.30	4	4	3.45	4
Diptera	Sciaridae	Detritivore	CSIRO (1991)	19	0.98	10	3	0.04	3
Diptera	Sphaeroceridae	Detritivore	CSIRO (1991)	5	0.76	4	-	-	-
Hemiptera	Tropiduchidae	Herbivore	CSIRO (1991)	10	44.55	6	2	2.23	2

Hymenoptera	Vespidae	Predator	Goulet and Huber (1993)	3	20.14	2	-	-	-
Lepidoptera	Tineidae	Omnivore	CSIRO (1991)	8	5.17	6	-	-	-
Polydesmida	Pyrgodesmidae	Detritivore	CSIRO (1991)	5	9.02	4	-	-	-
Hymenoptera	Ceraphronidae	Parasite	Goulet and Huber (1993)	5	0.10	4	27	0.72	17
Neuroptera	Chrysopidae	Predator	CSIRO (1991)	-	-	-	1	6.05	1
Lepidoptera	Gelechiidae	Herbivore	Stehr (2005)	-	-	-	3	2.06	3
Lepidoptera	Gracillariidae	Herbivore	CSIRO (1991)	-	-	-	12	3.57	11
Orthoptera	Gryllacrididae	Herbivore	CSIRO (1991)	-	-	-	4	325.74	3
Coleoptera	Phalacridae	Fungivore	CSIRO (1991)	1	0.30	1	2	0.60	2
Araneae	Pholcidae	Predator	Jocqué and Dippenaar-Schoeman (2006)	-	-	-	1	2.31	1
Diptera	Chloropidae	Detritivore	CSIRO (1991)	8	0.75	6	2	0.29	2
Diptera	Mycetophilidae	Fungivore	McAlpine (1981)	1	0.80	1	1	0.14	1
Araneae	Philodromidae	Predator	Jocqué and Dippenaar-Schoeman (2006)	-	-	-	1	30.76	1
Coleoptera	Carabidae	Predator	CSIRO (1991)	1	4.04	1	2	4.17	2
Diptera	Lauxaniiidae	Detritivore	CSIRO (1991)	5	2.72	4	-	-	-
Hemiptera	Meenoplidae	Herbivore	CSIRO (1991)	2	2.18	2	-	-	-
Hemiptera	Pseudococcidae	Herbivore	Ben-Dov (1994)	1	0.02	1	-	-	-
Hymenoptera	Ichneumonidae	Parasite	Goulet and Huber (1993)	16	5.72	12	1	2.02	1
Lepidoptera	Acanthopteroctetidae	Herbivore	CSIRO (1991)	1	6.09	1	-	-	-
Lepidoptera	Geometridae	Herbivore	Stehr (2005)	6	5.79	5	-	-	-
Hemiptera	Hydrometridae	Predator	CSIRO (1991)	-	-	-	2	17.94	2
Hemiptera	Derbidae	Herbivore	CSIRO (1991)	3	5.25	3	-	-	-
Hemiptera	Eriosomatidae	Herbivore	CSIRO (1991)	-	-	-	3	0.21	3
Diptera	Muscidae	Omnivore	CSIRO (1991)	3	0.61	3	3	1.31	3
Diptera	Deuterophlebiidae	Detritivore	Courtney 1997	-	-	-	1	0.01	1
Araneae	Dysderidae	Predator	CSIRO (1991)	-	-	-	1	5.79	1
Hemiptera	Reduviidae	Predator	CSIRO (1991)	5	34.31	5	5	606.11	4
Orthoptera	Tridactylidae	Herbivore	CSIRO (1991)	2	13.35	2	6	39.30	5
Hymenoptera	Bethylidae	Omnivore	CSIRO (1991)	-	-	-	3	1.86	3
Blattodea	Blaberidae	Detritivore	CSIRO (1991)	1	0.77	1	9	32.00	8
Diptera	Dolichopodidae	Predator	McAlpine (1981)	1	1.27	1	6	0.55	5

Diptera	Calliphoridae	Herbivore	CSIRO (1991)	2	1.19	1	-	-	-
Thysanoptera	Merothripidae	Detritivore	CSIRO (1991)	-	-	-	2	0.14	2
Lepidoptera	Noctuidae	Herbivore	Stehr (2005)	2	1.25	2	4	4.19	3
Hemiptera	Ricaniidae	Herbivore	CSIRO (1991)	-	-	-	1	0.26	1
Blattodea	Blattidae	Detritivore	CSIRO (1991)	1	5.42	1	8	177.08	8
Psocoptera	Pachytroctidae	Detritivore	Gruner (2004)	-	-	-	4	0.15	4
Diptera	Clusiidae	Detritivore	CSIRO (1991)	1	0.55	1	-	-	-
Ephemeroptera	Baetidae	Detritivore	CSIRO (1991)	3	4.52	3	1	1.86	1
Hymenoptera	Chalcididae	Parasite	Goulet and Huber (1993)	-	-	-	3	1.17	3
Hymenoptera	Platygastridae	Parasite	Goulet and Huber (1993)	2	0.05	2	1	0.03	1
Hemiptera	Schizopteridae	Predator	Reagan and Waide (1996)	-	-	-	6	0.52	6
Hemiptera	Coreidae	Herbivore	Schuh and Slater (1995)	1	41.75	1	-	-	-
Hymenoptera	Tiphiidae	Parasite	Goulet and Huber (1993)	2	4.76	1	-	-	-
Coleoptera	Chrysomelidae	Herbivore	CSIRO (1991)	4	30.50	4	3	4.45	3
Hemiptera	Tingidae	Herbivore	CSIRO (1991)	1	0.06	1	3	0.20	3
Lepidoptera	Zygaenidae	Herbivore	CSIRO (1991)	1	8.82	1	-	-	-
Araneae	Pisauridae	Predator	CSIRO (1991)	1	91.93	1	-	-	-
Hemiptera	Aradidae	Herbivore	CSIRO (1991)	1	1.46	1	1	0.44	1
Araneae	Anapidae	Predator	Jocqué and Dippenaar-Schoeman (2006)	-	-	-	3	4.51	3
Diptera	Simuliidae	Herbivore	CSIRO (1991)	-	-	-	4	0.18	4
Hemiptera	Lophopidae	Herbivore	CSIRO (1991)	1	0.68	1	-	-	-
Hymenoptera	Figitidae	Omnivore	CSIRO (1991)	1	0.06	1	-	-	-
Coleoptera	Anthicidae	Detritivore	CSIRO (1991)	-	-	-	1	1.10	1
Hymenoptera	Cynipidae	Herbivore	Goulet and Huber (1993)	-	-	-	2	0.25	1
Araneae	Deinopidae	Predator	Jocqué and Dippenaar-Schoeman (2006)	-	-	-	1	22.25	1
Polyxenida	Polyxenidae	Detritivore	CSIRO (1991)	-	-	-	2	0.64	2
Diptera	Bibionidae	Detritivore	CSIRO (1991)	1	11.80	1	-	-	-
Diptera	Drosophilidae	Detritivore	McAlpine (1981)	1	0.69	1	-	-	-
Hemiptera	Flatidae	Herbivore	CSIRO (1991)	4	3.72	3	-	-	-
Hemiptera	Geocoridae	Predator	Schuh and Slater (1995)	2	1.53	2	-	-	-
Diptera	Chironomidae	Detritivore	McAlpine (1981)	2	0.44	1	1	0.06	1

Araneae	Ochyroceratidae	Predator	Jocqué and Dippenaar-Schoeman (2006)	-	-	-	1	0.90	1
Araneae	Mysmenidae	Predator	Jocqué and Dippenaar-Schoeman (2006)	1	0.08	1	-	-	-
Diptera	Opomyzidae	Detritivore	McAlpine (1981)	3	0.61	3	-	-	-
Hymenoptera	Mymarommatidae	Fungivore	Goulet and Huber (1993)	-	-	-	3	0.04	3
Isoptera	Termitidae	Detritivore	CSIRO (1991)	-	-	-	1	2.67	1
Lepidoptera	Tortricidae	Herbivore	CSIRO (1991)	1	0.32	1	-	-	-
Psocoptera	Lepidopsocidae	Detritivore	Gruner (2004)	-	-	-	3	0.69	3
Phasmatodea	Phasmatidae	Herbivore	Bedford (1978)	-	-	-	1	1220.85	1
Araneae	Ctenidae	Predator	Jocqué and Dippenaar-Schoeman (2006)	-	-	-	1	10.97	1
Psocoptera	Elipsocidae	Detritivore	Gruner (2004)	-	-	-	2	0.08	2
Hemiptera	Alydidae	Herbivore	CSIRO (1991)	-	-	-	2	56.01	1
Psocoptera	Liposcelidae	Detritivore	Gruner (2004)	-	-	-	6	0.02	3
Hemiptera	Pentatomidae	Herbivore	CSIRO (1991)	-	-	-	1	117.74	1
Araneae	Tetragnathidae	Predator	Jocqué and Dippenaar-Schoeman (2006)	-	-	-	1	1.46	1
Araneae	Gnaphosidae	Predator	Jocqué and Dippenaar-Schoeman (2006)	-	-	-	2	5.18	2
Hymenoptera	Sphecidae	Predator	CSIRO (1991)	-	-	-	1	13.89	1
Psocoptera	Ectopsocidae	Detritivore	Gruner (2004)	-	-	-	1	0.02	1
Araneae	Clubionidae	Predator	Jocqué and Dippenaar-Schoeman (2006)	-	-	-	1	7.74	1
Hymenoptera	Aphelinidae	Parasite	CSIRO (1991)	1	0.06	1	2	0.14	1
Araneae	Miturgidae	Predator	Jocqué and Dippenaar-Schoeman (2006)	-	-	-	1	2.26	1
Diptera	Pipunculidae	Parasite	McAlpine (1981)	2	2.92	1	-	-	-
Diptera	Scatopsidae	Detritivore	McAlpine (1981)	1	0.06	1	-	-	-
Psocoptera	Psyllipsocidae	Detritivore	Gruner (2004)	-	-	-	2	1.91	2
Coleoptera	Curculionidae	Detritivore	CSIRO (1991)	1	0.50	1	-	-	-
Coleoptera	Anthribidae	Fungivore	CSIRO (1991)	2	0.58	1	-	-	-
Phthiraptera	Trichodectidae	Parasite	CSIRO (1991)	1	0.01	1	-	-	-

Appendix Table 11: Biotic data (first year; 56 plots). S_{spp} = species richness, N = abundance, B = body mass, S_{fam} = family richness, LL inv. = leaf-litter invertebrates, HL inv. = herb-layer invertebrates.

PlotID	Bird S_{spp}	Bird N	Bird B	LL inv. S_{fam}	LL inv. N	LL inv. B	HL inv. S_{fam}	HL inv. N	HL inv. B
1	4	10	243.42	10	42	42.95	5	6	64.52
2	6	13	765.44	9	20	288.91	11	24	161.83
3	6	11	513.33	7	31	9.86	14	42	438.59
4	5	13	739.86	15	88	224.38	26	120	282.16
5	5	13	514.29	5	35	21.33	10	21	199.73
6	7	16	579.26	7	14	19.93	22	58	532.47
7	6	10	299.32	11	15	16.42	22	45	428.10
8	8	19	2132.60	3	11	6.96	6	6	2.14
9	7	17	1313.05	7	10	46.52	22	53	692.00
10	6	14	887.65	4	10	14.69	12	37	178.17
11	7	11	641.10	6	30	18.36	24	57	735.88
12	5	8	178.96	12	43	19.45	30	86	320.63
13	7	10	753.19	10	74	11.79	16	57	490.74
14	4	13	699.06	4	10	4.45	10	19	79.50
15	6	13	1692.13	16	49	47.05	17	32	1086.18
16	6	10	234.05	4	14	29.50	14	28	206.38
17	5	7	352.63	7	16	4.66	12	20	513.22
18	3	15	459.39	5	6	9.14	12	22	103.47
19	6	11	1723.40	3	38	33.80	15	31	14.18
20	6	10	430.03	5	11	28.16	21	49	766.57
21	5	11	653.14	8	26	37.92	21	77	1268.84
22	5	11	444.66	3	15	42.93	5	13	6.64
23	5	11	506.14	16	33	13.55	25	91	669.98
24	5	9	298.20	6	97	123.11	20	65	707.65
25	6	15	1251.86	2	7	42.22	12	31	489.17
26	4	9	213.43	15	68	55.71	16	53	1965.31
27	7	10	967.46	10	115	269.54	21	56	409.00
28	3	9	304.58	7	217	253.83	4	8	89.81
29	7	13	720.56	14	26	17.11	24	52	394.15
30	5	10	115.40	3	3	0.72	12	16	22.44
31	2	6	76.20	4	9	6.35	12	17	1393.20
32	4	9	176.610	7	13	12.55	17	42	1087.27
33	4	8	716.70	4	9	18.57	20	81	384.49
34	3	6	221.240	17	27	58.33	15	28	507.55
35	2	4	73.37	12	44	26.66	11	49	588.41
36	4	6	117.91	5	52	48.07	21	37	480.15
37	8	15	754.06	3	226	265.06	16	79	502.26
38	4	12	234.39	13	62	21.62	24	69	307.77
39	7	17	1000.52	9	35	58.51	10	22	532.30
40	3	7	123.12	7	11	10.16	9	19	43.07
41	2	5	120.88	5	16	7.13	12	49	1159.40
42	4	6	276.35	3	10	3.89	17	39	228.19
43	6	10	305.30	9	16	25.02	24	97	975.05
44	5	9	615.08	5	33	45.23	17	26	105.81
45	6	16	1154.07	11	31	176.93	18	43	248.05
46	3	4	222.70	10	11	21.36	14	44	1007.57
47	3	4	93.55	14	115	31.61	15	67	1444.66
48	5	8	373.52	2	8	4.64	11	32	186.84
49	1	3	169.80	4	10	11.36	17	69	631.61
50	2	5	197.72	22	104	40.46	12	40	569.62
51	5	10	644.46	10	15	11.73	24	77	141.15
52	6	9	519.56	6	31	12.68	16	48	987.91
53	3	10	264.12	14	44	190.47	9	12	228.82
54	3	8	376.63	6	12	45.79	16	25	424.78
55	2	11	131.80	8	43	29.74	14	21	42.52
56	4	10	812.85	16	77	40.40	15	26	106.60

Appendix Table 12: Species/family numbers, beta diversity and Simpson index for plants, birds and leaf-litter invertebrates in forest, oil palm and the experimental site EFForTS-BEE.

		Forest	Oil palm	EFForTS-BEE
Plants	species richness (total)		78	53
	mean species richness (±SD)		32.25 ± 13.05	25.25 ± 6.9
Birds	species richness (total)	30	9	6
	Mean species richness (±SD)	11 ± 4.08	4.5 ± 1.29	2.25 ± 1.5
	mean α- diversity (±SD)	0.87 ± 0.05	0.58 ± 0.07	0.34 ± 0.4
Leaf-litter invertebrates	family richness (total)	47	25	16
	mean family richness	19.5 ± 7.94	7.5 ± 8.35	5.5 ± 2.38
	mean α- diversity (±SD)	0.65 ± 0.06	0.47 ± 0.26	0.52 ± 0.23

Appendix figures

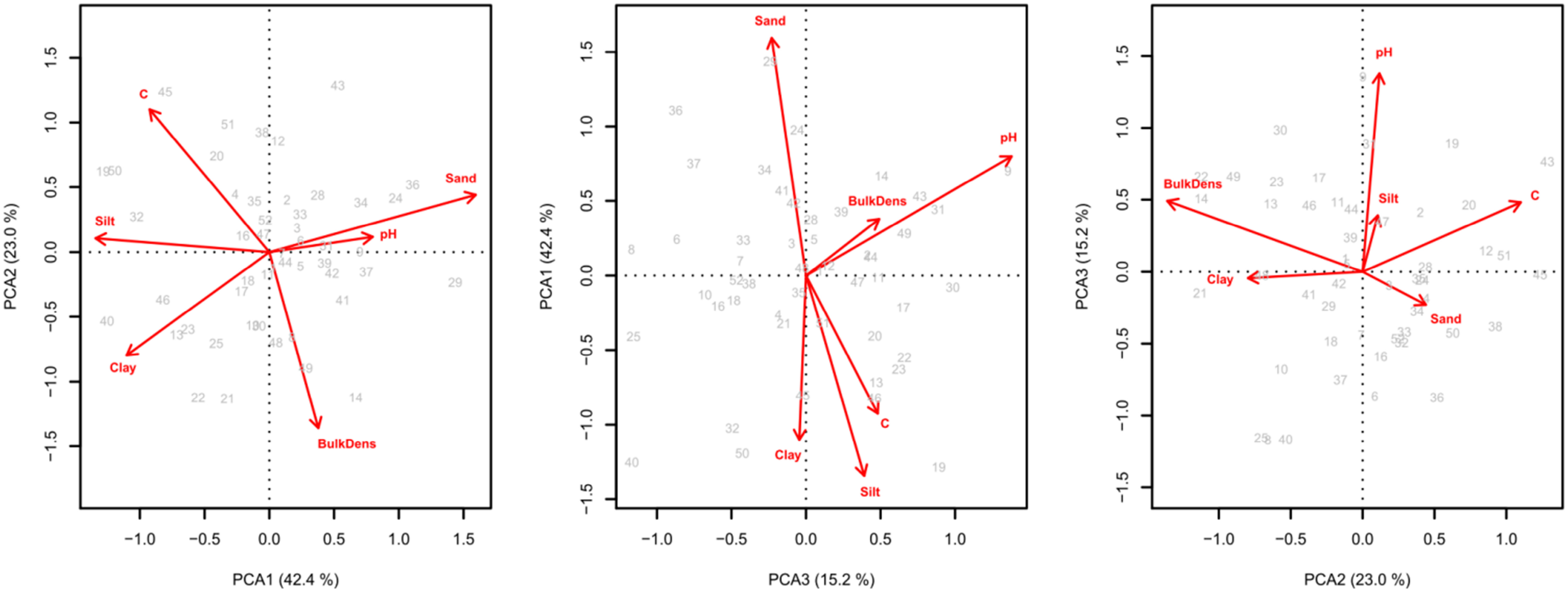

Appendix Figure 1: Principal component analysis of the soil data.

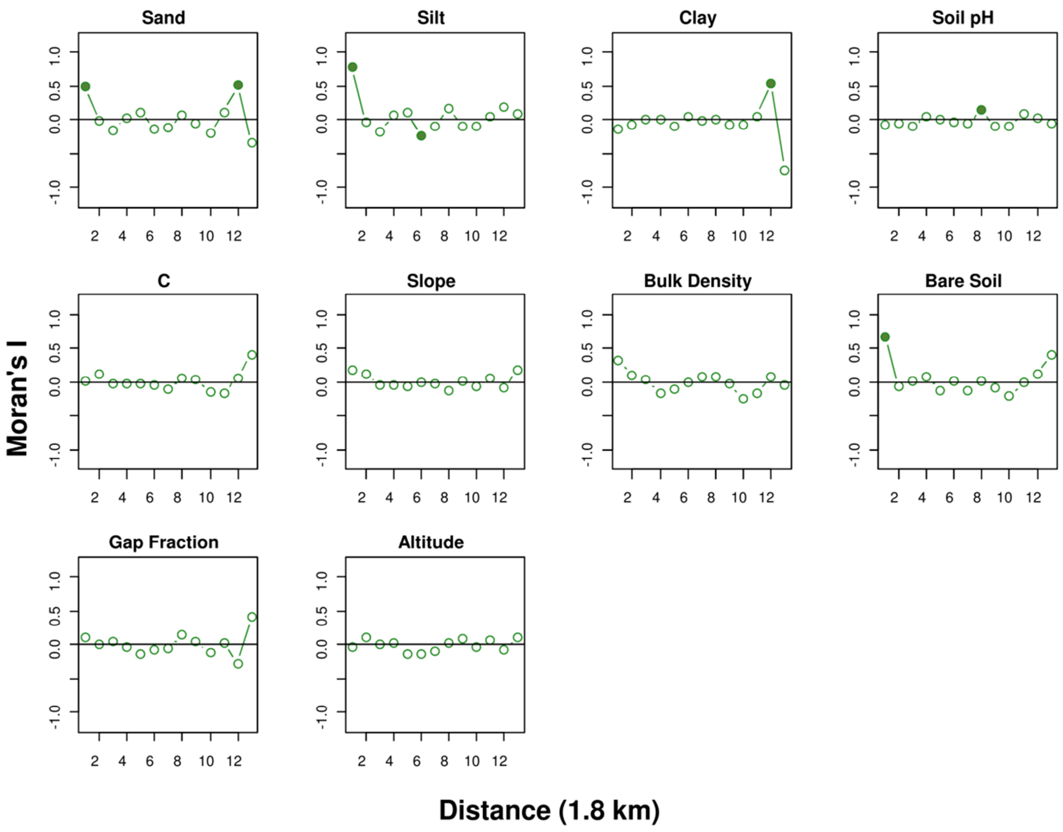

Appendix Figure 2: Spatial correlogram to check for spatial autocorrelation of the site-condition variables. Moran's I is given as a function of distance. Values significant at a nominal (two-sided) 5%-level are represented by filled circles and non-significant values by open circles.

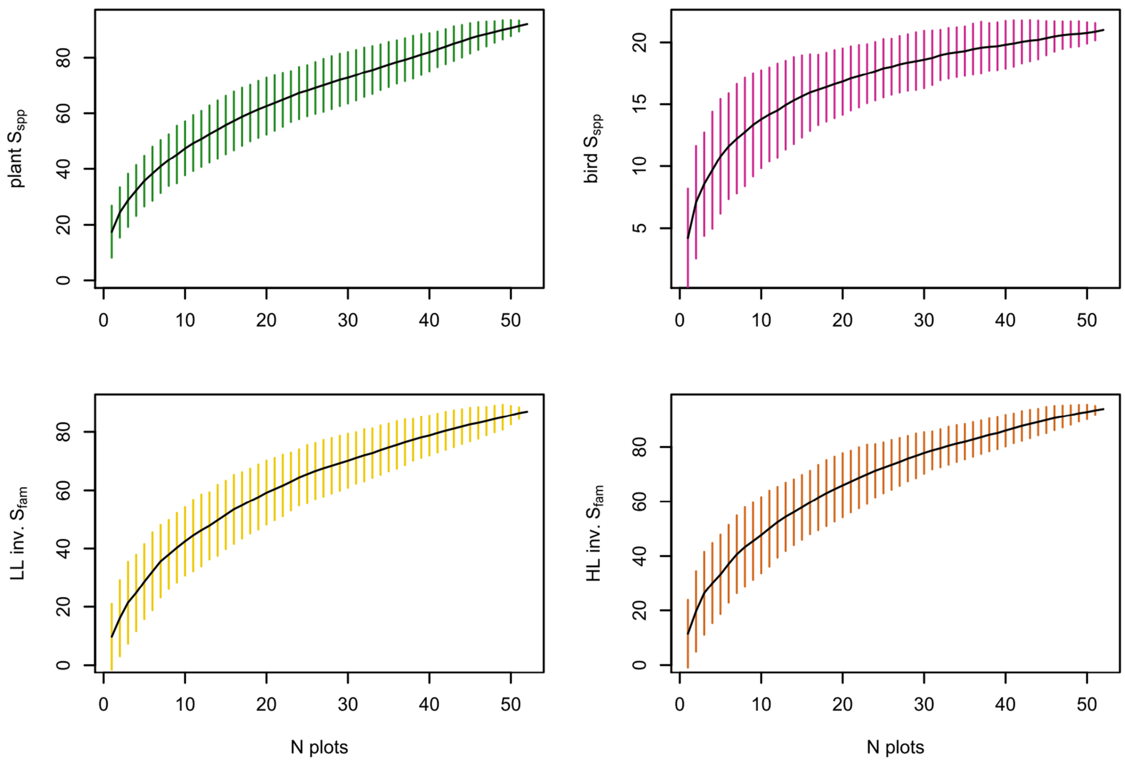

Appendix Figure 3: Species accumulation curves for plant and bird species (S_{spp}) as well as for families (S_{fam}) of leaf-litter (LL) and herb-layer (HL) invertebrates (inv.).

Supplementary references

Bedford, G. O. (1978). Biology and ecology of the phasmatodea. *Annual Review of Entomology* 23, 125–149. doi:10.1146/annurev.en.23.010178.001013.

Ben-Dov, Y. (1994). *A systematic catalogue of the mealybugs of the world: (Insecta; Homoptera; Coccoidea; Pseudococcidae and Putoidae) with data on geographical distribution, host plants, biology and economic importance*. Intercept Ltd.

Beukema, H., Danielsen, F., Vincent, G., Hardiwinoto, S., and Andel, J. (2007). Plant and bird diversity in rubber agroforests in the lowlands of Sumatra, Indonesia. *Agroforestry Systems* 70, 217–242. doi:10.1007/s10457-007-9037-x.

Capinera, J. L. (2008). *Encyclopedia of Entomology*. Springer.

Clarke, R. Q. S. (1973). *Handbooks for the identification of British insects. Coleoptera Heteroceridae*. London: Royal Entomological Society.

Colloff, M. J., Hastings, A. M., Spier, F., and Devonshire, J. (2005). Centipedes of Australia. *CSIRO Entomology and Australian Biological Resources Study*. Available at: http://www.ento.csiro.au/biology/.

Costa, C., Vanin, S. A., and Ide, S. (1995). Larvae of Neotropical Coleoptera 22. description of adults and immatures of *Lagrioida nortoni* sp. N., and bionomics (Coleoptera, Tenebrionoidea, Anthicidae). *Iheringia Serie Zoologia* 78, 113–126.

CSIRO (1991). *The insects of Australia : a textbook for students and research workers*. 2nd ed. Commonwealth Scientific and Industrial Research Organization.

CSIRO (2013). What bug is that - the guide to Australian insect families. *Commonwealth Scientific and Industrial Research Organization*. Available at: anic.ento.csiro.au/insectfamilies/.

David, J.-F. (2009). Ecology of millipedes (Diplopoda) in the context of global change. *Soil Organisms* 81, 719–733.

Edwards, R. (1996). Estimating live spider weight using preserved specimens. *Journal of Arachnology* 24, 161–166.

Eisenbeis, G. (2006). "Biology of soil invertebrates," in *Intestinal microorganisms of termites and other invertebrates*, eds. H. König and A. Varma (Springer), 3–53.

Gepp, J., and Hölzel, H. (1989). *Ameisenlöwen und Ameisenjungfern - Myrmeleonidae*. Neue Brehm Bücherei.

Goulet, H., and Huber, J. T. eds. (1993). *Hymenoptera of the world. An identification guide to families*. Canada Communication Group.

Gowing, G., and Recher, H. F. (1984). Length-weight relationships for invertebrates from forests in south-eastern New South Wales. *Austral Ecology* 9, 5–8. doi:10.1111/j.1442-9993.1984.tb01612.x.

Gruner, D. S. (2003). Regressions of length and width to predict arthropod biomass in the Hawaiian Islands. *Pacific Science* 57, 325–336. doi:10.1353/psc.2003.0021.

Gruner, D. S. (2004). Attenuation of top-down and bottom-up forces in a complex terrestrial community. *Ecology* 85, 3010–3022. doi:10.1890/04-0020.

Harvey, M. (2015). Schizomids. *Australasian Arachnological Society*. Available at:

http://www.australasian-arachnology.org/arachnology/schizomida/.

Höfer, H., and Ott, R. (2009). Estimating biomass of Neotropical spiders and other arachnids (Araneae, Opiliones, Pseudoscorpiones, Ricinulei) by mass-length regressions. *Journal of Arachnology* 37, 160–169. doi:10.1636/T08-21.1.

IUCN (2015). Red List of threatened species. *International Union for Conservation of Nature*. Available at: www.iucnredlist.org.

Jocqué, R., and Dippenaar-Schoeman, A. S. (2006). *Spider families of the world*. Musée Royal de l'Afrique Centrale.

Johnson, M., and Strong, A. (2000). Length-weight relationships of Jamaican arthropods. *Entomological News* 111, 270–281.

Kocarek, P., Holusa, J., Grucmanova, S., and Musiolek, D. (2011). Biology of *Tetrix bolivari* (Orthoptera: Tetrigidae). *Central European Journal of Biology* 6, 531–544. doi:10.2478/s11535-011-0023-y.

Lang, A., Krooss, S., and Stumpf, H. (1997). Mass-length relationships of epigeal arthropod predators in arable land (Araneae, Chilopoda, Coleoptera). *Pedobiologia* 41, 327–333.

McAlpine, J. F. (1981). *Manual of Nearctic Diptera*. Canadian Government Publishing Centre.

Mercer, R., Gabriel, A., Barendse, J., Marshall, D., and Chown, S. (2001). Invertebrate body sizes from Marion Island. *Antarctic Science* 13, 135–143. doi:10.1017/S0954102001000219.

Pappas, J. (2001). "*Coturnix chinensis*" (Online). *Animal Diversity Web*. Available at: http://animaldiversity.org/accounts/Coturnix_chinensis/ [Accessed December 15, 2015].

Pappas, M. L., Xanthis, C., Samaras, K., Koveos, D. S., and Broufas, G. D. (2013). Potential of the predatory mite *Phytoseius finitimus* (Acari: Phytoseiidae) to feed and reproduce on greenhouse pests. *Experimental and Applied Acarology* 61, 387–401. doi:10.1007/s10493-013-9711-9.

Pinto-da-Rocha, R., Machado, G., and Giribet, G. (2007). *Harvestmen: the biology of Opiliones*. Harvard University Press.

Reagan, D. P., and Waide, R. B. eds. (1996). *The food web of a tropical rain forest*. University of Chicago Press.

Robson, C. (2015). *Birds of South-East Asia: concise edition*. London: Bloomsbury, Christopher Helm.

Sample, B. E., Cooper, R. J., Greer, R. D., and Whitmore, R. C. (1993). Estimation of insect biomass by length and width. *American Midland Naturalist* 129, 234. doi:10.2307/2426503.

Schowalter, T. D. (2009). *Insect ecology: an ecosytem approach*. Elsevier.

Schuh, R. T., and Slater, J. A. (1995). *True bugs of the world (Hemiptera: Heteroptera)*. Cornell University Press.

Stehr, F. W. (2005). *Immature insects*. Kendall Hunt Publishing.

Thiollay, J.-M. (1995). The role of traditional agroforests in the conservation of rain forest bird diversity in Sumatra. *Conservation Biology* 9, 335–353. doi:10.1046/j.1523-1739.1995.9020335.x.

Wardhaugh, C. W. (2013). Estimation of biomass from body length and width for tropical rainforest canopy invertebrates. *Australian Journal of Entomology* 52, 291–298. doi:10.1111/aen.12032.

Wilman, H., Belmaker, J., Simpson, J., de la Rosa, C., Rivadeneira, M. M., and Jetz, W. (2014). EltonTraits 1.0: species-level foraging attributes of the world's birds and mammals. *Ecology* 95, 2027–2027. doi:10.1890/13-1917.1.

Wilson, S. W., Mitter, C., Denno, R. F., and Wilson, M. R. (1994). "Evolutionary patterns of host plant use by delphacid planthoppers and their relatives," in *Planthoppers: Their Ecology and Management*, eds. R. F. Denno and T. J. Perfect (New York: Chapman and Hall), 7–113.

Yosef, R., International Shrike Working Group, and de Juana, E. (2015). "Long-tailed Shrike (*Lanius schach*)," in *Handbook of the Birds of the World Alive*, eds. J. del Hoyo, Elliott, A., J. Sargatal, D. A. Christie, and E. de Juana (Barcelona: Lynx Edicions).

Zimmer, M. (2002). Nutrition in terrestrial isopods (Isopoda: Oniscidea): an evolutionary-ecological approach. *Biological reviews of the Cambridge Philosophical Society* 77, 455–493. doi:10.1017/s1464793102005912.
